# FusionSeq: a modular framework for finding gene fusions by analyzing paired-end RNA-sequencing data

**DOI:** 10.1186/gb-2010-11-10-r104

**Published:** 2010-10-21

**Authors:** Andrea Sboner, Lukas Habegger, Dorothee Pflueger, Stephane Terry, David Z Chen, Joel S Rozowsky, Ashutosh K Tewari, Naoki Kitabayashi, Benjamin J Moss, Mark S Chee, Francesca Demichelis, Mark A Rubin, Mark B Gerstein

**Affiliations:** 1 Program in Computational Biology and Bioinformatics, Yale University, 300 George Street, New Haven, CT 06511, USA; 2 Molecular Biophysics and Biochemistry Department, Yale University, 260 Whitney Avenue, New Haven, CT 06520, USA; 3 Department of Pathology and Laboratory Medicine, Weill Cornell Medical College, 1300 York Avenue, New York, NY 10065, USA; 4 Department of Urology, Weill Cornell Medical College, 525 East 68th Street, New York, NY 10065, USA; 5 Prognosys Biosciences, Inc., 505 Coast Blvd South, La Jolla, CA 92037, USA; 6 Institute for Computational Biomedicine, Weill Cornell Medical College, 1305 York Avenue, New York, NY 10065, USA; 7 Department of Computer Science, Yale University, 51 Prospect Street, New Haven, CT 06511, USA

## Abstract

We have developed FusionSeq to identify fusion transcripts from paired-end RNA-sequencing. FusionSeq includes filters to remove spurious candidate fusions with artifacts, such as misalignment or random pairing of transcript fragments, and it ranks candidates according to several statistics. It also has a module to identify exact sequences at breakpoint junctions. FusionSeq detected known and novel fusions in a specially sequenced calibration data set, including eight cancers with and without known rearrangements.

## Background

Deep sequencing approaches applied to transcriptome profiling (RNA-Seq) are dramatically impacting our understanding of the extent and complexity of eukaryotic transcription [1-4]. RNA-Seq provides a more accurate measurement of expression levels of genes and more information about alternative splicing of their isoforms compared to other chip-based methods [1,4-10].

Large international consortia, such as the ENCODE project [11] and the modENCODE project [12], are exploiting this technology to obtain a better picture of the transcriptome. More recently, RNA-Seq was applied to the identification of fusion transcripts, where mRNAs from two different genes are joined together [13-17]. Although the role of these chimeric transcripts is not fully understood, some studies have shown that they might be implicated in cancer [18,19]. Also, a fusion transcript may indicate an underlying genomic rearrangement between the two genes. Such gene fusions are thought to drive molecular events, such as in chronic myelogenous leukemia, which is defined by the reciprocal translocation between chromosome 9 and 22 leading to a chimeric fusion oncogene (*BCR-ABL1*) encoding a tyrosine kinase that is constitutively active.

Most gene fusions reported in the past have been attributed to hematological cancers [20-22]. Recently, recurrent fusions between the transmembrane protease serine 2 (*TMPRSS2*) gene and members of the ETS family of transcription factors (mainly the v-ets erythroblastosis virus E26 oncogene homolog (avian), *ERG*, and the ets variant 1, *ETV1*) were reported in prostate cancer [23]. Other epithelial tumors, such as lung and breast cancer, also harbor translocations [24-26].

Compared to DNA sequencing, RNA-Seq seems to have less requirements in terms of overall coverage, since it aims at sequencing only the regions of the genome that are transcribed and spliced into mature mRNA, which current estimates set at about 2 to 6%. However, this apparent advantage of RNA-Seq in practice is not so straightforward. Indeed, determining the depth of sequencing needed to completely assess the extent of transcription in complex organisms is complicated by the high dynamic range of gene expression, the presence of alternatively spliced transcripts, and the biological condition of the transcriptome, that is, cell types or environmental conditions [2].

### State-of-the-art

RNA-Seq can be used effectively to detect fusion transcripts. Maher *et al. *[13] discovered novel fusion transcripts using single-end reads of various lengths. This approach nominated multiple candidates such as *SLC45A3-ELK4*, which was independently confirmed as a common 'read-through' transcript identified in prostate cancer (that is, fusion transcripts resulting from two nearby genes without any genomic rearrangement [19]). This and other non-genomic events of adjacent or neighboring genes appear to be common. Maher *et al. *showed in principle how to use RNA-Seq to discover fusion transcripts. They used two single-end sequencing platforms, which is rather infeasible in terms of both cost and labor efforts [13]. Since then, paired-end (PE) RNA-Seq has been introduced and has received broader attention for transcriptome profiling, bringing with it great potential to accelerate fusion discoveries [14,15].

The concept of sequencing both ends of a fragment, either cDNA or genomic DNA, was introduced in the context of the identification of structural variants [27-31]. Such events are among the basic mechanisms generating fusion transcripts. The main advantage of PE reads is that the connectivity information between the sequenced ends is available. PE sequencing is thus the obvious method to employ for identifying fusion transcripts. In a path-breaking study, Maher *et al. *[15] analyzed PE RNA-Seq data and demonstrated the feasibility of this technology to confirm known gene fusions and identify novel fusion transcripts. Their study also confirmed the need for a systematic analysis accounting for computational complexity and statistical significance. The method proposed, however, relies on the distance between the two ends of a transcript fragment (insert size). This idea, inspired by structural variant analysis, cannot be directly translated to the transcriptome analysis in order to obtain an accurate description of all the occurring events. The main reason is the complexity of the transcription, and in particular the splicing of introns, that can lead to read pairs spanning several exons, as we describe in detail later.

Two more recent studies focus on the identification of novel splice junctions from RNA-Seq data [32,33]. This problem is related to the discovery of fusion transcripts because, in principle, a 'splice junction' can indeed join two different genes and thus suggest a fusion event. Although these methods can, in principle, be applied to the discovery of fusion transcripts, they mainly focus on the mapping of the reads. They do not analyze the impact of artifacts independent from the mapping procedure on the detection of fusion transcripts, such as the random pairing of transcript fragments during sample preparation (see Materials and methods). These tools also do not provide a means to summarize the results of the detection of potential fusion transcripts. Finally, the experimenter would not have the flexibility of using other mapping tools that may provide complementary information. Specifically, SplitSeek is currently available only for AB/SOLiD [33].

To address these issues, we developed FusionSeq, a novel computational suite whose aim is to detect candidate fusion transcripts by analyzing PE RNA-Seq data [34]. FusionSeq is mapping-independent as much as possible, such that it is not bound to a single platform or mapping approach. It accounts for several sources of errors in order to provide a high-confidence list of fusion candidates, which are also scored by using several statistics to prioritize experimental validation. FusionSeq also includes tools to summarize and present its results integrated into a web browser. Furthermore, we sequenced an appropriate data set to calibrate this approach, comprising mostly human prostate cancer tissues with and without known fusion events.

## Results and discussion

### Mapping the reads

The first step when dealing with next-generation sequencing is the alignment of the reads against known reference sequences. Here the main challenge is how to map millions of reads in a computationally efficient way. Several alignment tools have been developed and, since this research field is quite active, it is likely that improved or new tools will be introduced. In addition, a variety of mapping strategies can be employed. As an example, a splice junction library may be employed along with the reference genome to identify reads bridging exons. Our goal is to develop a method that is independent as much as possible from mapping strategies and alignment tools. As a test, we tried a variety of alignment tools and approaches, all yielding consistent results, thus demonstrating the robustness of FusionSeq (Additional file 1). For simplicity, we here report the results obtained by mapping the reads to the genome with ELAND, the standard program supplied with the Illumina platform (see Materials and methods). Table 1 reports the results of the mapping (details in Additional file 1).

**Table 1:** Results of the alignment

Sample ID	Type	Known fusion type	Read size (nt)	Total number of PE reads	Mapped PE reads	Percentage of mapped PE reads
106_T	PCa	TMPRSS2-ERG	51	7,239,733	4,723,941	65.25%
1700_D	PCa	TMPRSS2-ERG	51	12,435,299	7,629,273	61.35%
580_B	PCa	TMPRSS2-ERG	36	18,134,550	7,690,673	42.41%
99_T	PCa	NDRG1-ERG	36	2,844,879	1,515,444	53.27%
2621_D	PCa	SLC45A3-ERG	54	22,079,700	11,899,984	53.90%
1043_D	PCa	No known fusions	51	3,003,305	1,898,332	63.21%
NCI-H660	PCa cell line	TMPRSS2-ERG	51	6,512,688	4,120,365	63.27%
GM12878	Lymphoblastoid cell line	No known fusions	54	44,829,991	20,676,159	46.12%

Total number of PE reads, number of mapped PE reads and the percentage mapped are reported, Note that the number of single-end reads is double the number of PE reads. PCa, prostate cancer.

### Overall modular framework

**Figure 1:**
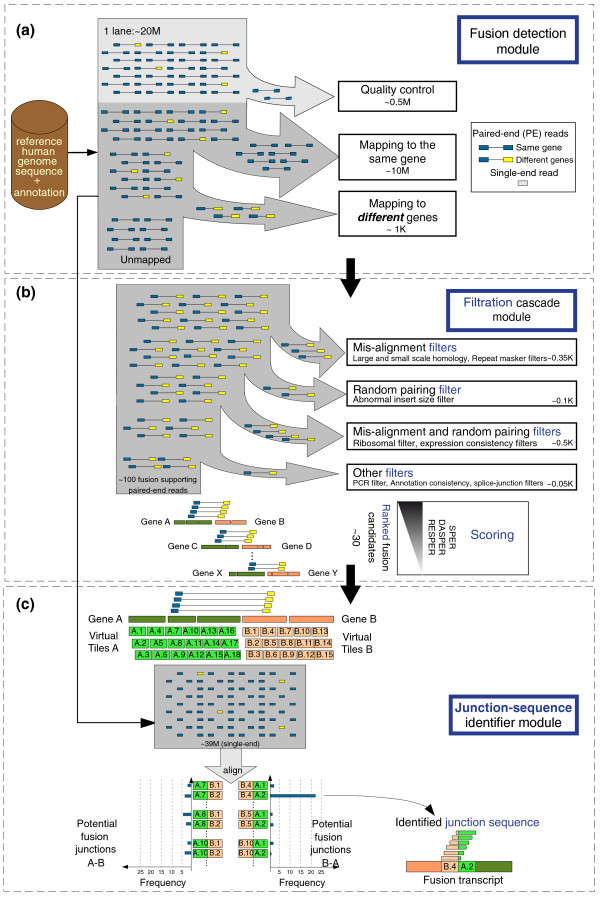
**Schematic of FusionSeq**. **(a) **The PE reads are processed to identify potential fusion candidates. Poor quality reads are discarded at first, and the remaining PE reads are aligned to the reference human genome (hg18). The reads are compared to the annotation set (UCSC Known Genes) in order to classify them as belonging to the same gene or to different genes. Those aligned to two different genes are then selected as potential fusion candidates. All good quality single-end reads are also stored for the identification of the sequence of the junction. **(b) **The filtration cascade module analyzes the candidates and removes those that have high sequence homology between the two genes or a higher insert size compared to the transcriptome norm. Additional filters are employed to remove candidates due to random pairing and misalignment as well as PCR artifacts and annotation inconsistencies. The high-confidence list of candidates is then scored and processed to find the sequence of the junction. **(c)**. The junction-sequence identifier detects the actual sequence at the breakpoints by constructing a fusion junction library. It first covers the regions of the potential breakpoint of each gene with 'tiles' 1 nt apart, and then creates all possible combinations, considering both orientation of the fusion, namely gene A upstream of gene B and *vice versa*. All single-end reads are then aligned to the fusion junction library and the junction with the highest support is identified as the sequence of the fusion transcript junction. *DASPER*, difference between the observed and analytically calculated expected SPER; *RESPER*, ratio of empirically computed SPERs; *SPER*, supportive PE reads.

The overall schematic of our approach is depicted in Figure 1. It consists of three modules.

#### Module 1: fusion transcript detection

This module only assumes that the PE reads have been aligned and their location is known. It identifies the set of candidate fusions from the mapped sequence reads. Conceptually, it consists of three steps (Figure 1a): step 1, poor quality reads are removed; step 2, PE reads that map to the same gene are considered part of the normal transcriptome; step 3, PE reads that map to different genes are selected as potential candidate fusion transcripts; also, reads that do not align anywhere are stored for the computational validation of the candidates and for determining the sequence of the junctions. Note that the mapping of the reads can occur anywhere within a gene: exons, introns or splice junctions.

We employ a reference annotation set (University of California Santa Cruz (UCSC) Known Genes [35]) and classify each single-end of a PE read into different categories depending on what parts of the gene it is mapped to: exon, intron, splice junction or boundary. The latter case corresponds to reads that might be mapped to the genomic boundary of an exon - for example, in the case of a retained intron or when pre-mRNA is sequenced.

#### Module 2: filtration cascade

Several types of noise can introduce artifacts at any stage of the sequencing and analysis process. Hence, we developed a number of different filters to reduce the problem of artificial chimeric transcripts (Figure 1b). Additional filters, more specific to the reference annotation set employed, are described in Additional file 1.

##### Three misalignment filters

The reads can be mapped to a different location on the genome compared to where they were generated, mainly because of the sequence similarity of regions in the genome (paralogs, pseudogenes, repetitive elements). Indeed, it is possible that single nucleotide polymorphisms (SNPs), RNA editing, or errors in the base caller can lead to misalignment of one of the ends resulting in artificial chimeric transcripts. This issue is particularly relevant in the intermediate range of sequencing depth (1 million to 100 million reads), which FusionSeq has been designed for. We devised three filters to deal with this issue of sequence similarity, briefly described hereafter (see Materials and methods for detail).

###### Large scale sequence similarity filter

If the two genes of a candidate fusion transcript are paralogous, they are discarded because of this homology potentially causing a misalignment. We use TreeFam to identify these candidates and remove them from the list [36,37].

###### Small scale sequence similarity filter

The above filter seeks broad similarities between two transcripts. However, it may be possible that there is high similarity between small regions within the two genes where the reads actually map. To identify these cases, for each of the candidate chimeric transcripts, the reads aligned to one gene are searched for sequence similarity against the corresponding partner. If high similarity is found, the pair is removed (Materials and methods).

###### Repetitive regions filter

Some reads may be aligned to repetitive regions in the genome due to the low sequence complexity of those regions and may result in artificial fusion candidates. We thus remove reads mapped to those regions (Materials and methods).

##### Random pairing of transcript fragments: abnormal insert size filter

The filters described so far deal with computationally generated artifacts. However, some artifacts can be intrinsic to the experimental protocol. Library preparation typically requires the fragmentation of the cDNA. This may result in the generation of random chimeric transcripts when inefficient A-tailing may lead to the ligation of random cDNA molecules [38]. This issue affects more highly expressed genes. The abnormal insert size filter addresses this problem by exploiting the fact that the transcript fragments have approximately the same size because a size-selection step is typically part of the experimental protocol. We could filter the set of candidate fusion transcripts by selecting those paired reads having an insert size - that is, the distance between the two mapped reads - comparable to the fragment size and by excluding those with a much higher insert size, somewhat resembling the approach for determining DNA structural variants [27,39-41]. However, this approach is based on the fact that the alignment of genomic PE reads to the genome reflects its linearity, where any deviation from this 'nominal' insert size will be considered abnormal (Figure S1a in Additional file 1). These approaches cannot be directly translated to RNA-Seq analysis because of at least three additional layers of complexity: the splicing mechanism of the transcription; the genome of the individual, which contains some differences from the reference genome; and the cancer genome of the same individual, which can include additional somatic variations (Figure S1b in Additional file 1).

**Figure 2:**
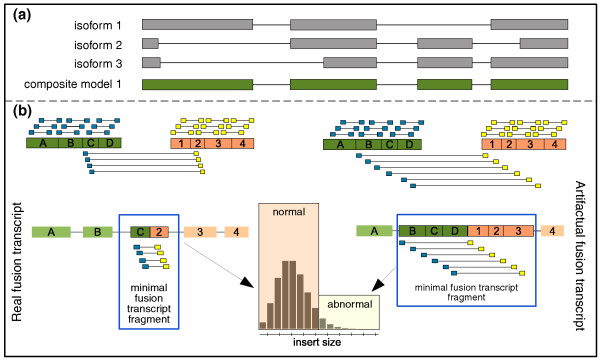
**Abnormal insert-size principle applied to transcriptome data**. The composite model of a gene is created via the union of the exonic nucleotides from all its isoforms. By using the composite model, we can exploit the abnormal insert-size principle. A minimal fusion transcript fragment is created by connecting the regions of the two genes joined by PE reads. Subsequently, the insert-size of these chimeric PE reads is computed and compared to the insert-size distribution of PE reads in the normal transcriptome. The higher insert-size compared to the transcriptome norm would suggest an artifact since it may be due to the random joining of fragments during library generation.

We devised a method to address some of these issues and still make use of this concept to identify true chimeric transcripts. We first introduce the concept of the 'composite model' of a gene - that is, the union of all exons from all known isoforms of a gene - and then we define the 'minimal fusion transcript fragment' (Figure 2). This is generated by using all PE reads bridging the two different genes. It is important to note that in the case of a real fusion transcript, we can only identify the region around the fusion junction. Reads generated by a fusion transcript that are distant from the junction will be assigned to one gene or the other. For a real chimeric transcript, the minimal fusion transcript fragment will thus capture the region around the breakpoint and the insert-size distribution computed on it will be similar to the insert size distribution of normal transcripts. Conversely, for an artifactual chimeric transcript, paired reads would randomly join the two genes from all different parts (Figure 2b, right-hand side). The minimal fusion transcript fragment would be bigger than the expected fragment. Hence, the insert-size distribution computed on this minimal fusion transcript fragment will be higher than that of normal transcripts, that is, abnormal. The normal insert-size distribution can be estimated from the data by using the composite models of all genes (see Materials and methods).

##### Two filters for the combination of misalignments and random pairing

An additional complication is the possibility that random pairing and misalignment occur together. Highly expressed genes may generate transcript fragments that randomly join with another gene. In addition, misalignment can affect the correct identification of the genes involved in this random pairing. This is particularly challenging because only a fraction of the reads from random pairing will be misaligned; specifically, those with high similarity to another region of the genome. This would result in PE reads bridging relatively small regions that can escape the abnormal insert size filter. Hence, we devised two additional filters: one comparing the candidates to the typically highly expressed ribosomal genes, and the other assessing the consistency of the expression levels of the individual genes of a chimeric transcript (see Materials and methods).

##### PCR filter

Most library preparations also require a PCR amplification step. This may lead to potentially artifactual fusion candidates when the same read is over-represented, yielding to a 'spike-in-like' signal, that is, a narrow signal with a high peak. To reduce this effect, we filter candidates that have chimeric reads piling up in a small region (see Materials and methods).

#### Module 3: junction sequence identifier

After the identification of high-quality candidate fusion transcripts, we can seek the overall support of those candidates taking advantage of the pool of all single-end reads. This process also allows the identification of the exact sequence of the fusion transcript junction. The knowledge of the actual junction sequence has many uses. First, it can help to identify the actual regions that are connected in the fusion transcript. Second, it helps in subsequent experimental validation, such as by RT-PCR. Finally, it can provide additional evidence for the fusion transcript or can be used to rule out artifacts.

In order to identify the junction sequence, we build a 'fusion junction library' and align all single-end reads to this library (Figure 1c). To be computationally efficient, we first identify the regions where the potential breakpoints are using the information from the PE reads bridging the two genes. The exact size of the regions bears greatly on the resulting complexity of the potential fusion transcript and the computational power (see Materials and methods). Then, we cover these regions with 'tiles' that are spaced 1 nt apart and, finally, we generate the fusion junction library by creating all pair-wise connections between these tiles. The rationale is that the correct junction sequence will correspond to one of these connected tiles and that there will be full-length single-end reads that will align to that sequence (see Materials and methods).

### Scoring the candidates

Although FusionSeq filters out many spurious fusion candidates, some may still be present, especially random chimeric transcripts generated during sample preparation. Hence, candidates are scored based on their likelihood to be real, allowing prioritization of validation experiments. The first obvious measure is simply the number of inter-transcript PE reads (*m_i_*) normalized by the total number of mapped PE reads (*N_mapped_*), similarly to RPKM (reads per kilobase of exon model per million mapped reads) for measuring gene expression [3]. This is expressed per million mapped reads and called *SPER *for '*s*upportive PE reads'. For the *i*-th candidate:

$$SPER_{i}=\frac{m_{i}}{N_{mapped}}\cdot {\text{10}}^{\text{6}}$$

This measure gives an indication of the abundance of the fusion transcript. However, to assess whether a given *SPER *is 'high' enough, we compare it with two 'expected' values: one is calculated analytically and the other empirically. The first quantity is *DASPER *(the difference between the observed and analytically calculated expected *SPER*), indicating how many (normalized) inter-transcript PE reads we observe more than expectation. The analytically calculated expected *SPER *(<*SPER *>) is based on the observation that if two ends were randomly joined, the probability that this occurs for gene A and gene B is proportional to the product of the probability that the two single-ends of the pair are mapped to gene A and gene B (see Materials and methods). This scoring method takes into account fusion transcripts that might have been generated during sample preparation from highly expressed genes. Obviously, the higher *DASPER *is, the more likely the fusion candidate is real.

The second measure is *RESPER *(the ratio of empirically computed *SPER*s). The rationale for this measure is the comparison of the observed *SPER *with the *SPER*s of the other candidates. We expect a real fusion transcript to be supported by a higher number of reads compared to the artifactual chimeric transcripts (see Materials and methods). This quantity, contrary to *DASPER*, is independent of the fragment size, thus more suitable for comparisons across samples. While *RESPER *is useful, it suffers in comparison to *DASPER *if a sample has several real fusions.

In summary, by computing these quantities, we can 'demote' fusion candidates that may result from random joining of highly expressed genes (*DASPER*), and select those candidates that 'stand out' compared to the others (*RESPER*), thus providing a high-confidence ranked list of candidates.

### Classifying the candidates

FusionSeq provides a list of potential fusion candidates that are automatically classified into different categories depending on the genes that are involved [13]: (1) inter-chromosomal - two genes on different chromosomes; (2) intra-chromosomal - two genes on the same chromosomes. The latter can be further subclassified as: (2a) read-through candidates if the two genes are close neighbors on the genomes, that is, if no other gene is present between them; (2b) *cis *candidates - similar to read-through events, but the two genes are on different strands.

Several read-through events have been reported in the literature, although their role remains unclear [42]. This may also be an effect of the pervasive transcription of the genome. Indeed, when considering primary transcripts, more than 90% of the nucleotides of the human genome are transcribed [11]. Although the RNA-Seq protocol requires a poly-A selection step, it may occur that pre-mRNA fragments with stretches of adenosines are still selected and sequenced.

### FusionSeq applied to prostate cancer samples

In order to develop and calibrate FusionSeq, we selected a set of prostate cancer tissues harboring the common *TMPRSS2-ERG *fusion, others with less common fusions (*SLC45A3-ERG*, *NDRG1-ERG*) and prostate cancers with no evidence of known ETS fusions. We also sequenced a prostate cancer cell line with the *TMPRSS2-ERG *fusion (NCI-H660) and a lymphoblastoid cell line (GM12878) that was selected for the HapMap project and employed by the ENCODE project as controls. This normal cell line is not expected to have gene fusions (Table 1). Overall, FusionSeq takes about 2 hours to analyze 20 million mapped reads. More details about the computational complexity are discussed in Materials and methods.

#### Fusion candidates

The application of FusionSeq to the above samples resulted in the identification of 12 fusion candidates, on average, per sample with *SPER *greater than 1 (range 0 to 25). Considering the top candidate for each sample, the average *SPER *is 13.99 for those with known *ERG *rearrangements and 3.09 for those without known fusions (Table 2; Table S1 in Additional file 1). The vast majority of candidate fusions are intra-chromosomal - they occur between genes that are on the same chromosome - with the majority being read-through events (Table S1 in Additional file 1).

**Table 2:** *SPER*, *DASPER*, and *RESPER *for the top candidates with *DASPER *> 0 and *RESPER *> 1 across all prostate cancer tissue samples

Type	ID	Fusion candidate	*SPER*	*DASPER*	*RESPER*
**Intra**	**580_B**	**TMPRSS2-ERG**	**36.54**	**36.53**	**14.31**
**Intra**	**1700_D**	**TMPRSS2-ERG**	**19.66**	**19.63**	**8.79**
**Intra**	**106_T**	**TMPRSS2-ERG**	**10.16**	**10.11**	**3.97**
**Inter**	**2621_D**	**SLC45A3-ERG**	**4.29**	**4.15**	**3.56**
**Inter**	**1700_D**	**ERG-GMPR**	**4.59**	**4.59**	**2.05**
Read-through	1700_D	SLC16A8-BAIAP2L2	4.33	4.33	1.93
*Read-through*	*106_T*	*AK094188-AK311452*	*4.87*	*4.87*	*1.9*
*Read-through*	*1700_D*	*ZNF473-FLJ26850*	*3.54*	*3.54*	*1.58*
*Read-through*	*580_B*	*ZNF577-FLJ26850*	*4.03*	*4.03*	*1.58*
*Read-through*	*1043_D*	*ZNF577-ZNF649*	*5.79*	*5.79*	*1.55*
*Read-through*	*1700_D*	*CAMTA2-INCA1*	*3.01*	*3.01*	*1.35*
Inter	1700_D	EEF1D-HDAC5	2.88	2.84	1.29
*Read-through*	*1043_D*	*FLJ00248-LRCH4*	*4.74*	*4.74*	*1.27*
*Read-through*	*1700_D*	*VMAC-CAPS*	*2.62*	*2.62*	*1.17*
*Read-through*	*106_T*	*FLJ00248-LRCH4*	*2.96*	*2.96*	*1.16*
Cis	1043_D	AX747861-FLI1	4.21	4.21	1.13
*Read-through*	*106_T*	*TAGLN-AK126420*	*2.75*	*2.75*	*1.07*
**Inter**	**580_B**	**PIGU-ALG5**	**2.73**	**2.73**	**1.07**
**Inter**	**99_T**	**NDRG1-ERG**	**7.26**	**7.15**	**1.02**

Cell lines are reported in Table S1 in Additional file 1. Entries in bold are known gene fusions, and those in italics read-through events confirmed either experimentally or via additional evidence, such as ESTs or mRNAs from GenBank.

The most common fusion, *TMPRSS2-ERG*, is ranked at the top of the list. The other known fusions between *ERG *and other 5' partners, namely *SLC45A3 *and *NDRG1*, are also included in the top candidates. The remaining candidates appear to be read-through events, including *ZNF649-ZNF577 *(Table 2).

Although the candidates are ranked by *RESPER*, it is worth noting that the *TMPRSS2-ERG *fusion has high values for both *SPER *and *DASPER*, as expected. These statistics are almost equivalent for the top candidates; however, they substantially differ in the case of artifacts given by highly expressed genes (Tables S1, S3 and S5 in Additional file 1), suggesting the effectiveness of *DASPER *in identifying those cases. Indicatively, *DASPER *and *RESPER *values greater than 1 seem to conservatively select for true chimeric events, with 16 out of 19 candidates (84%) being either experimentally confirmed or with EST evidence.

We find a second candidate fusion transcript involving *ERG *and *GMPR *in sample 1700_D in addition to *TMPRSS2-ERG*. By analyzing the regions that are connected, it seems that the exons not involved in the *TMPRSS2-ERG *fusion are linked to *GMPR*, suggesting that *ERG *undergoes a balanced translocation. This novel finding was experimentally validated (Figure S2 in Additional file 1). Another novel finding is the fusion transcript involving *PIGU *and *ALG5 *that was also experimentally confirmed [43]. Finally, there is one *cis *candidate including *AX747861 *and *FLI1*, which may suggest some complex rearrangement (Materials and methods). However, from EST data there is evidence that this may correspond to a single *FLI1 *transcript, thus suggesting an artifact caused by the annotation set (Figure S3 in Additional file 1). Although FusionSeq can properly handle such cases with the annotation filters (Additional file 1), we report it here as an example of how the framework can be employed to refine the search of candidate fusion transcripts and help the experimenter screen this list.

#### Effects of the filters

**Figure 3:**
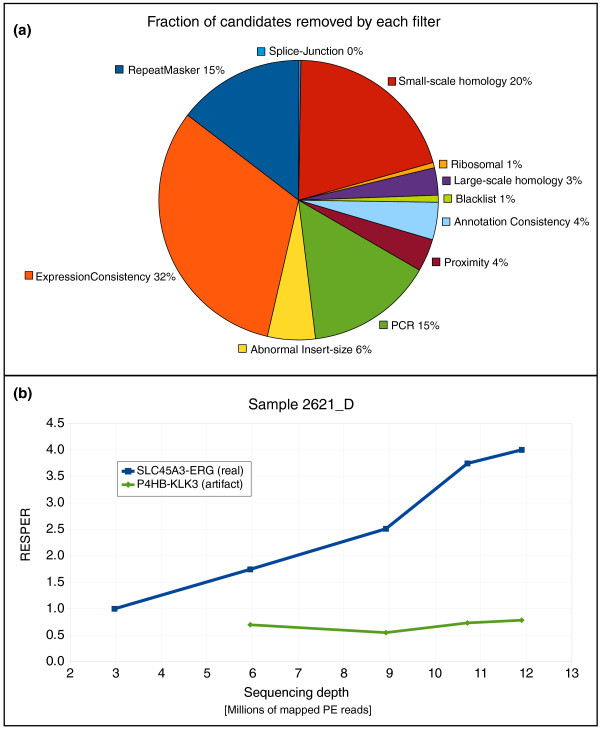
**Filtration cascade module**. **(a) **The average percentage of candidates identified by the fusion detection module that are removed by each filter is reported. The labels also depict the order the filters have been applied in this case (counter-clockwise starting from the RepeatMasker filter), but it is worth noting that the order of the application of the filters does not affect the final list of candidates. **(b) ***RESPER *(ratio of empirically computed SPERs) versus depth of sequencing. The plot shows the *RESPER *values for *SLC45A3-ERG*, a real fusion transcript, and *P4HB-KLK3*, an artifact likely created by the random pairing due to the high expression of KLK3 at different sequencing depths.

The application of the filters reduced the number of candidates identified by the fusion detection module. Out of a total of 7,342 candidates, only 133 candidates passed all the filters, a reduction of 98% (average number of identified candidates per sample = 917.75, range [451 to 1,618]; average number of candidates per sample after filtering = 16.63, range [4 to 41]). In Figure 3a, we summarize the effect of the filters. Each filter reduces the number of potential candidates to some extent, indicating that they address these issues. We experimentally verified that some of the candidates filtered out or with negative *DASPER *are artifactual (Table S6 in Additional file 1).

#### Sequencing depth and detection of fusion candidates

We investigated the effect of the number of mapped reads on the detection of fusion transcripts. We randomly sampled fractions of mapped reads from sample 2621_D, and applied FusionSeq to the reduced data sets (see Materials and methods). The top candidate is always *SLC45A3-ERG *with an increasing *RESPER*, as expected (Figure 3b). That *RESPER *increases with increasing sequencing depth is an indicator that the real fusion transcript stands out compared to the background. Although the number of fusion candidates increases as well, the *DASPER *for the majority of other candidates is negative, suggesting that they are artifacts (Table S1 in Additional file 1).

#### *TMPRSS2-ERG *fusion-positive prostate cancer tissues

**Figure 4:**
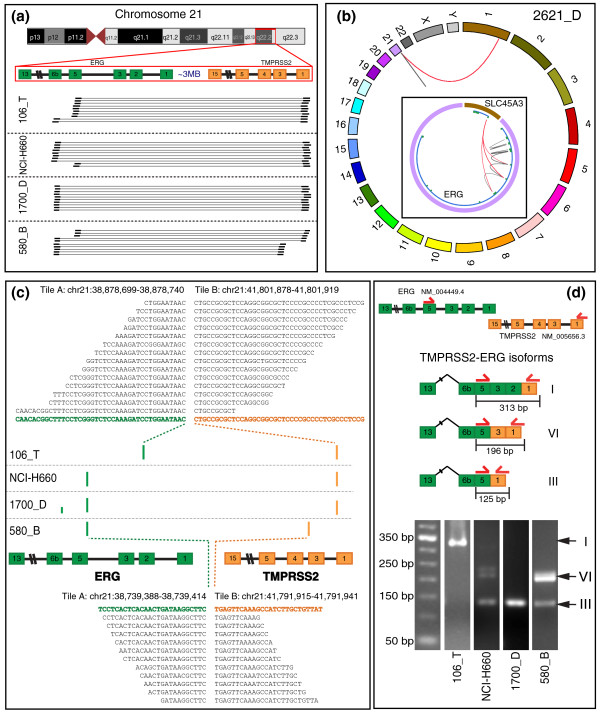
**Results of FusionSeq**. **(a) **A subset of the PE reads connecting *TMPRSS2 *and *ERG *are shown for four samples (106_T, NCI-H660, 1700_D, 580_B). **(b) **PE reads connecting *ERG *and *SLC45A3 *for sample 2621_D. The outer circle reports all chromosomes, whereas the inset shows only the region of *ERG *and *SLC45A3*. The gray lines depict the intra-transcript PE reads, whereas the red ones represent the inter-transcript PE reads. Note that for illustration purposes, only the inter-transcript reads are shown for *SLC45A3*. The inset also depicts the composite model (blue line) and its exons (green boxes). **(c) **Results of the junction-sequence identifier. The location of the breakpoints for the four samples with the *TMPRSS2-ERG *fusion are reported as bars (not to scale). Moreover, the sequence of the junctions as well as a subset of the aligned reads for two samples is reported (106_T, 580_B). **(d) **The locations of the PCR primers used for the validation are depicted as red arrows. The isoforms consist of *TMPRSS2 *and *ERG *exons fused to form different exon combinations as depicted schematically. For both samples NCI-H660 and 1700_D, isoform III is detected, whereas, for samples 106_T and 580_B, isoforms I and VI are determined, respectively (Table S7 in Additional file 1) [46,56]. The transcript isoforms were validated by a PCR assay for each sample separately (gel images). A 50-nt length standard (lane 1) is shown here for the determination of the approximate fragment size. The identity of the PCR products was validated by Sanger sequencing.

For all the *TMPRSS2-ERG*-positive prostate cancer tissues, FusionSeq always detects this fusion transcript at the top of the list (Table S1 in Additional file 1). Figure 4a shows the PE reads bridging the two genes for the three tissue samples and the cell line harboring the fusion for the entire region between *TMPRSS2 *and *ERG*. It is worth noting that the regions connected by the PE reads are different across the samples, suggesting the presence of different *TMPRSS2-ERG *isoforms.

#### Exon expression

The expression of a fusion transcript should also be reflected in the intensity of the signal at the exon level. Specifically, if a fusion transcript does not include some exons of the 'wild-type' gene, the expression of those excluded exons should be lower compared to that of exons that are part of the fusion transcript. This observation was originally reported by Tomlins *et al. *[23] using a standard exon walking experiment and has been confirmed using exon arrays [44].

**Figure 5:**
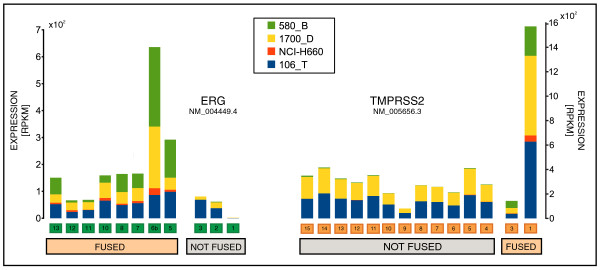
**Expression values of the exons of *TMPRSS2 *and *ERG***. The RPKM values computed on each exon of *ERG *(isoform NM_004449.4) and *TMPRSS2 *(isoform NM_005656.3) are shown as stacked bars for the four samples with *TMPRSS2-ERG *fusion. For illustration purposes, the exons included in the most common fusion isoforms are labeled as 'FUSED'.

For illustration purposes, Figure 5 shows the expression values (RPKM) for the exons of *ERG *and *TMPRSS2*. It is common that the expression of *ERG *is driven by its fusion with a 5' partner. Hence, we can expect that the major expression signal is due to the fusion transcript. Indeed, the expression signal of the exons involved in the fusion transcript is higher than that of the region excluded. A similar conclusion is obtained when looking at *TMPRSS2*.

#### Junction-sequence identification analysis

Figure 4c shows the results of the junction-sequence identifier module for the four samples with *TMPRSS2-ERG *fusion. The main breakpoints are detected for both *TMPRSS2 *and *ERG*. This allows the determination of the correct fusion isoform, which was experimentally validated with RT-PCR (Figure 4d). By taking a closer look at the junction-sequence identification results, a second potential breakpoint for sample 1700_D can be detected, albeit with much fewer number of reads (5 compared to 320 for the main breakpoint; Figure S4a in Additional file 1). The reads supporting it are uniformly distributed across the junction, suggesting that it is a real breakpoint and that multiple fusion variants are present. This finding has been validated with RT-PCR using a primer specific to this junction (Figure S4b in Additional file 1).

#### *ERG*-rearranged cases with different 5' partners

We analyzed two other *ERG*-rearranged cases where the 5' partner of *ERG *is different from *TMPRSS2*. We previously reported the discovery of a novel rearrangement between *ERG *and *NDRG1 *for sample 99_T, resulting from the focused analysis of PE RNA-Seq restricted to the specific region of *ERG *[14]. With the current method that performs a genome-wide analysis, we confirmed the *NDRG1-ERG *fusion transcript as the top candidate (Table 2). Furthermore, we applied FusionSeq to another *ERG*-rearranged sample, 2621_D, identifying *SLC45A3-ERG *as top candidate (Table 2, Figure 4b).

#### *ERG *rearranged-negative case and normal cell line

When applied to the sample without known fusion transcripts (1043_D), FusionSeq detected only a few candidates, the top being the read-through event between *ZNF577 *and *ZNF649*, which is common in all prostate tissues analyzed here and has been already documented [13]. For the GM12878 cell line, it is noteworthy that, despite having more than 20 million mapped PE reads, none of the few candidates (*n *= 4) have a *SPER *higher than 0.3, as expected being a normal cell line (Table S1 in Additional file 1). The read-through event with positive *DASPER *appears to be a mis-annotation of the untranslated regions (UTRs; *BC110369-BC080605*), whereas the inter-chromosomal candidates have a negative *DASPER*, suggesting that they may be due to random chimeric pairing. Indeed, one of the genes involved is a highly expressed gene, *ACTG1*, with an RPKM >232,000 [3]. Furthermore, the junction-sequence identifier analysis does not yield any result.

### Simulation results

In addition to experimental evidence, we also performed a simulation study to assess FusionSeq performance. We employed the GM12878 cell line as an estimate of the background because it is not expected to harbor any fusion transcripts. We randomly generated inter-transcript reads, thus simulating the presence of fusion transcripts, and added these PE reads to the pool of the actual PE reads of the GM12878 cell line data (see Additional file 1 for details). The results showed that a *DASPER *score greater than 1 achieves high sensitivity (0.80) even if the fusion transcript is expressed at half the rate of the 'wild-type' allele (F = 0.5) with an area under the receiver operating characteristic (ROC) curve (AUC) higher than 0.95 (Figure S5 in Additional file 1).

## Conclusions

Gene fusions have been considered the key molecular event in leukemias, lymphomas, and some soft tissue tumors (that is, sarcomas). With the 2005 discovery of common recurrent gene fusions in prostate cancer, there exists a strong likelihood that recurrent gene fusions are present in common epithelial cancers [23]. Numerous studies have now confirmed that approximately 50% of prostate cancers harbor a recurrent fusion between *TMPRSS2 *and *ERG *or *ETV1 *[45]. In an attempt to identify these fusion events, we employed PE RNA-Seq technology exploiting the connectivity information of the two ends of transcript fragments. As is the case for other applications of deep sequencing, considerations of computational complexity and statistical significance are mandatory.

### FusionSeq: a modular framework

In the current study, we describe FusionSeq, a novel computational and statistical framework to identify fusion transcripts by analyzing PE RNA-Seq data. This framework consists of three modules: a fusion transcript detection module; a filtration cascade module, which is composed of a set of filters that remove different types of artifacts and rank the candidates by different scores; and a junction-sequence identifier module, which detects the actual sequence of the fusion junction.

Among the advantages of our method is the decoupling of the alignment approach from the identification of candidate fusion transcripts. Indeed, we developed FusionSeq to be independent from the alignment tool and the mapping strategy as much as possible. Other methods proposed that could potentially identify fusion transcripts require a particular choice of the mapping tool or platform and do not provide any considerations about artifactual fusion transcripts generated by the sequencing protocol [32,33]. To this end, we have developed a set of filters to remove artifactual candidates generated by several sources of errors (see Materials and methods), which are particularly relevant in the intermediate range of sequencing depth (1 million to 100 million reads). It is likely that with higher coverage those issues will impact the analysis less since one can use the statistics of the higher coverage to overcome errors.

Of further interest is also the ability of this method to identify the sequence of the junction of the fusion transcript using the full read length. This valuable information allowed us to detect and then experimentally confirm the simultaneous presence of multiple fusion isoforms within a single cancer tissue. Moreover, it enables the experimentalist to narrow the genomic region to look at for the subsequent validation of the fusion candidate. All validated fusions in this data set have breakpoints lying at the exon boundary. This might indicate that, in case of genomic rearrangement, the splicing machinery is still active and removes the intronic regions harboring the actual genomic breakpoints. Hence, we speculate that insertions or deletions that typically occur at genomic breakpoints might not affect the junction of the fusion transcript.

### Scoring the candidates

One of the novel features introduced by FusionSeq is the computation of scores to assign a 'confidence value' to the fusion candidates. We propose a classification and scoring approach to prioritize the selection of candidates for experimental validation (see Materials and methods).

We envision that researchers seeking gene fusions can use this tool to focus their efforts on the candidates with the top scores. Validation typically includes seeking confirmation of the putative fusion sequence using standard PCR assays and traditional sequencing as well as exploring for a corresponding genomic rearrangement at the DNA level using such approaches as fluorescence *in situ *hybridization (FISH).

### Sample set

One important aspect of this study is that we tested FusionSeq on data generated from cancer samples derived from human tumors and not only cell lines. Clearly these types of samples are more challenging given their heterogeneity as they may include tumor, stromal, and endothelial cells. We have used a set of prostate cancer samples with and without the *TMPRSS2-ERG *fusion transcript to calibrate FusionSeq. This well-characterized gene fusion was not only detected where present, but the junction sequence identifier also detected the correct junctions, thus enabling the determination of the specific isoform variants. Moreover, we observed that one sample had multiple variants.

Understanding the complexity of isoform splicing in cancer may not only add insight into biology, but may also provide useful prognostic information as it has been suggested that some *TMPRSS2-ERG *isoforms play a distinct role in prostate cancer development [46,47].

Furthermore, FusionSeq identified two novel events (*ERG-GMPR *and *PIGU-ALG5*), demonstrating that our procedure is able to find new fusions in addition to well-characterized ones.

### Reporting the results

FusionSeq also includes tools to access and display the results of the analysis through a web-browser by seamlessly integrating the UCSC Genome Browser. Moreover, to display inter-chromosomal events, which is currently not possible in the UCSC Genome Browser, we developed SeqViz, a visualization tool based on Circos [48], an open source software particularly suited for this purpose (see Materials and methods). These web-based interface tools enable the user to quickly access the information provided by FusionSeq, an aspect that greatly increases its applicability in comparison to other related tools [32,33].

### Future directions

Although we demonstrated the feasibility of this approach using several cancer tumor samples, there are some limitations to the current approach. The fusion transcript detection module is based on a gene annotation set that provides the information of the genes and their isoforms. Although the framework is flexible and the choice of which annotation to use is left to the user, the identification of the candidate fusion is of course limited to this set. We employed the UCSC Known Genes set, which contains 66,803 isoforms. We believe that this is a reasonable choice and that the use of a different annotation set would not dramatically change our results.

Although FusionSeq is independent from the mapping strategy adopted, it is likely that different mapping approaches would make use of the filtration cascade differently. As an example, if the alignment procedure explicitly excludes repetitive regions, the filter using RepeatMasker will impact on the final list of candidates to a lesser extent. This is why the modularity of FusionSeq allows the users to adapt the framework to their specific goals (Figure S9 in Additional file 1).

We anticipate that FusionSeq will benefit from the availability of longer sequence reads and deeper sequencing, with an increased ability to identify and score novel fusion events from RNA-Seq data.

## Materials and methods

### Prostate cancer selection and RNA extraction

All the prostate cancer samples were collected under an IRB (Institutional Review Board) approved protocol. Hematoxylin and eosin (H&E) slides were prepared from frozen tissue blocks and evaluated for cancer extent and tumor grade by the study pathologist (MAR). To ensure high purity of cancer cells and minimize benign tissue, tumor isolation was performed by first selecting for high-density cancer foci (< 10% stromal or other non-tumor tissue contamination) and then taking 1.5 mm biopsy cores from the frozen tissue block for RNA extraction using TRIzol Reagent (Invitrogen, Carlsbad, CA, USA). The RNA extract was then subjected to DNase treatment using a DNA-*free*™ Kit (Applied Biosystems/Ambion, Austin, TX, USA). The quality of RNA was assessed using the RNA 6000 Nano Kit on a Bioanalyzer 2100 (Agilent Technologies, Santa Clara, CA, USA). Up to 10 μg of RNA with RIN (RNA integrity number) ≥7 was determined suitable for sample preparation.

### Sample preparation

The samples were prepared in accordance with the Illumina RNA sample preparation protocol (Part # 1004898 Rev. A September 2008). Briefly, mRNAs were fragmented at elevated temperature using divalent cations and transcribed into cDNA, thereby generating a library of cDNA fragments. RNA-Seq adapters were then ligated to the blunt ends of the cDNA fragments. The library of cDNA fragments subsequently underwent a size-selection step in which cDNAs were first electrophoresed through a 2.5% agarose gel in TAE buffer. Then, the desired fragment size products (200 or 300 nt) were retrieved from the gel and subjected to PCR amplification using universal primer sites present at the end of the ligated adapters. The library was then subjected to quality control steps such as verification of fragment size and concentration measurements using the DNA 1000 Kit (Agilent Technologies) on an Agilent 2100 Bioanalyzer.

All samples were sequenced using one lane of an Illumina Genome Analyzer II (GAII) flowcell, except for GM12878 and 99_T, which were sequenced using two lanes. Since the experiments were performed over several months as Illumina introduced advances to the GAII platform, the total number of reads and the read length vary (Table 1). However, all samples were prepared following the same protocol.

### Validation of *TMPRSS2-ERG *fusion isoforms with PCR

Aliquots from the same RNA stock were used for both RNA-Seq and PCR validation by conventional reverse-transcription PCR. RNA was reverse transcribed using a High Capacity cDNA Reverse Transcription Kit (Applied Biosystems, Foster City, CA, USA). The *TMPRSS2-ERG *PCR was performed using Platinum *Taq *DNA Polymerase (Invitrogen) with 1 mM MgCl_2_, 0.1 μM of each primer (forward, *TMPRSS2 *exon 1 - TAGGCGCGAGCTAAGCAGGAG; reverse, *ERG *exon 5 - GTAGGCACACTCAAACAACGACTGG; as published by Tomlins *et al. *[23]) and 50 ng cDNA at an annealing temperature (Ta) of 63°C for 35 cycles and the PCR products were separated on a 2.5% agarose gel. For *TMPRSS2-ERG *isoform IV, the PCR was performed, using a reverse primer specifically designed for the detection of isoform IV (TGCATTCATCAGGAGAGTTCCTGC), under the same conditions but with Ta 55°C and 40 cycles. The obtained products were isolated from the gel using the MinElute™ Gel Extraction Kit (Qiagen, Valencia, CA, USA) and subsequently sent for Sanger sequencing at the Core facility of Cornell University (Ithaca, NY, USA).

### Mapping

We employed ELAND to map the PE reads against the Human Reference Genome (March 2006 Assembly - hg18). We allowed for up to two mismatches of the alignment and selected reads that passed the quality filter from ELAND. In case of pairs mapped to the same chromosome, we selected reads aligned to opposite strands. We also employed bowtie to map the reads to the human genome sequence [49]. Since bowtie does not allow PE reads to be mapped on different chromosomes, we adopted the following strategy: the two ends were mapped separately to the genome and the best alignment was selected among the top ten candidates in the case of mapping to multiple locations. Two mismatches were allowed for bowtie too. Then, the two ends were paired together, if both ends were aligned. Moreover, for comparison purposes, we mapped the reads to a splice junction and a ribosomal library in addition to the genome (see Additional file 1 for details).

### Filtration cascade

#### Large scale sequence similarity filter

Two paralogous genes resulting as fusion candidates are discarded because their homology can potentially cause a misalignment. We use TreeFam to identify and remove these candidates [36,37]. TreeFam is a database of phylogenetic trees of animal genes with the aim of providing a curated list of orthologs and paralogs.

#### Small scale sequence similarity filter

The small scale sequence similarity filter seeks broad similarities between two transcripts. However, it may be possible that there is high similarity between small regions within the two genes where the reads actually map. Hence, to search for similar sequences within the two candidate genes, we employ a two-step strategy. We first perform a fast search of the reads aligned to one gene against the full transcriptome, represented by all composite models, using bowtie [49]. If more than a user-defined threshold (default of 1%) of the reads map to one gene 'hit', the partner gene, the candidate is discarded. This approach removes candidates where the reads have high similarity, since bowtie allows up to three mismatches only. For those candidates not filtered out by this approach, a second, more refined comparison is performed. We align the reads mapped to one gene to its partner's sequence by using BLAT [50]. If the fraction of reads that have similarities to the corresponding partner is higher than a user-defined threshold (default of 1%), then the pair is discarded. In order to call a hit - that is, similarity to the partner gene - we require that at least 75% of the read has similarity to the corresponding gene.

#### Repetitive regions filter

Some reads may be aligned to repetitive regions in the genome due to the low sequence complexity of those regions, which may result in artificial fusion candidates. We thus remove reads mapped to repetitive regions using RepeatMasker to identify these regions [51,52].

#### Abnormal insert size filter

The PE RNA-Seq experimental protocol requires sequencing of the ends of cDNA molecules of a determined length: the fragment size. If we mapped those sequenced reads to the transcriptome (which we do not know exactly), we would obtain an insert-size distribution - the distance between the two reads - similar to the fragment size. However, since the reads are aligned to the reference genome, the insert-size distribution can be rather skewed (Figure S1b in Additional file 1). Using a splice junction library does not help in this context. Besides having potential biases given by the incomplete knowledge of the junctions, it cannot determine to which isoform the two ends belong. The composite model allows use of the concept of the insert-size also for RNA-Seq data (Figure 2a). The composite model is the union of all the exons from all known transcripts of a gene. This ensures that all exonic nucleotides are considered. The insert-size distribution computed using the composite model as reference should thus be comparable to the nominal fragment size selected during sample preparation (if there is more than one isoform, the insert size distribution computed with the composite model will be slightly shifted towards higher values because of the inclusion of all possible exonic regions in the composite model).

We then extend this concept to distinguish potentially real chimeric transcripts from artifactual ones. We generate a minimal fusion transcript fragment by using all the PE reads bridging two different genes (Figure 2b). The rationale is that the insert size distribution computed on this minimal fusion transcript fragment of a real chimeric transcript is similar to the insert size distribution of normal transcripts. This is because we expect inter-transcript PE reads to connect the regions around the junction only. Conversely, a fusion transcript generated by random chimeric pairing would have a rather long minimal fusion transcript because paired reads would randomly join different regions of the two genes. This, in turn, would yield a much higher insert-size distribution compared to that of the real case; that is, it would be abnormal.

Specifically, for each of the candidate chimeras, the insert-size distribution is computed using all paired reads mapping to the composite model of that gene: that is, the intra-transcript insert-size distribution. For this purpose, only reads that are fully contained within exons are considered. If a candidate has only intronic reads, this filter is not applied. Similarly, the 'anomalous' reads - reads that bridge two different genes - are first used to create a minimal fusion transcript (Figure 2b). Note that from the PE data we cannot determine the full fusion transcript, but only the region nearby the actual junction of the two genes, that is, the minimal fusion transcript fragment. Then, the insert-size distribution of the minimal fusion transcript is computed (inter-transcript insert-size distribution) and compared with the intra-transcript insert-size distribution. If the median of the inter-transcript insert-size distribution is much higher than the median of the intra-transcript insert-size distribution, it is likely due to misalignments. A *P*-value is computed by randomly sampling the intra-transcript insert-size distribution. Candidate fusion transcripts having a *P*-value lower than a user-defined cutoff are discarded as artifacts. Note that the candidates that are 'outliers' with respect to the intra-transcript insert-size distribution are discarded as artifacts, whereas, in the DNA context, they are kept as potential insertions or deletions (Figure S1a in Additional file 1).

For this analysis, we used a *P*-value cutoff of 0.01 (corresponding to approximately 2.5 standard deviations from the transcriptome norm) for all samples, except for 2621_D, for which we used a cutoff of 0.0001 because of the much tighter intra-transcript insert-size distribution given by the smaller fragment size compared to the other samples.

#### Ribosomal filter

The vast majority of transcripts in the cell are ribosomal RNA. Although the experimental protocol typically requires either selecting for non-ribosomal mRNA with polyA+ selection or depleting of ribosomal RNAs, this process is imperfect. This translates into a high abundance of ribosomal transcripts with a higher chance of generating random chimeras. If misalignment occurs too, this would result in artifactual candidates that appear to not involve ribosomal genes. Hence, this filter compares the reads of the candidates to the ribosomal genes sequence database using a more sensitive alignment tool such as BLAT [50]. If the reads align to ribosomal genes, the candidate is removed.

Specifically, in order to identify reads that bear similarity to ribosomal genes but were mapped to another region, we require a read to have more than 75% similarity to a ribosomal gene to count it as a hit. If more than 10% of reads map to the ribosomal library, the candidate is discarded (Additional file 1). Note that this issue, although related, is independent of the mapping strategy. Indeed, even if we employ a ribosomal library during the alignment phase, there still may be reads that, due to misalignment, will map best to other regions of the genome.

#### Expression consistency filter

Highly expressed genes give rise to the same issue that occurs with ribosomal genes. This filter compares the expression signal (that is, number of reads) generated by the chimeric reads to the signal of the individual genes. The rationale is that, in the case of a real fusion transcript, the two genes would be expressed at the same or higher levels than the 'chimeric' signal, whereas, in the case of an artifactual candidate, the signal would be generated only from the chimeric reads and the signal of the two individual genes would be much lower.

In more detail, the expression signal of the fusion candidate is computed by counting the number or inter-transcript, that is, chimeric, reads mapped and normalizing by the length of the region covered by those reads. The expression of the individual genes is computed as the number of reads normalized by the length of the transcripts. If the chimeric reads have a higher signal than that of the individual genes, the candidate is discarded.

#### PCR filter

To avoid artifacts resulting from the PCR amplification step, we require the reads supporting a candidate fusion transcript to independently cover at least *p *nucleotides (default *p *= 5) in addition to the read size on both ends, otherwise the candidate is discarded. This ensures that several instances of the transcripts were expressed in the cells. In the case of sufficient coverage, it is also possible to compute the entropy to identify these cases and remove them.

### Junction-sequence identifier module

The PE reads can identify the genes involved in a fusion transcript, but cannot directly determine the junction sequence because, typically, short read alignment tools do not allow gapped alignment for the single read. Hence, we developed this module to take advantage of the fast short read alignment tools and identify the sequence of the junction in an efficient way.

Let us assume we have some PE reads joining gene A with gene B, thus suggesting a fusion event between them. Those reads would connect regions around the junction. For each gene, we thus select the region that can include the junction sequence by first considering all exons that can be potentially involved in the junction as well as the intronic regions that are supported by chimeric PE reads. Those regions are extended considering the flanking 150 nucleotides. We then cover them with a set of 'tiles' that are spaced 1 nt apart and construct a fusion junction library by creating all pairwise junctions between these tiles. Since we do not know *a priori *what the specific form of the fusion transcript is, we create two libraries, one assuming gene A is upstream of gene B and the other assuming gene B is upstream of gene A (Figure 1c). This fusion junction library plays the same role as a canonical splice junction library: it enables the alignment of short reads, thus overcoming the need for a computationally expensive gapped alignment for reads bridging two exons or, as in this case, regions of different genes.

All the reads, including the non-mapped ones, are then mapped against this library. In this case we consider the two ends independently. The rationale is that the actual junction sequence will be described by a certain pair of tiles, and reads not previously mapped anywhere in the genome now can be aligned to this fusion junction. Moreover, reads that previously mapped with one or two mismatches to the reference genome may now map perfectly to the fusion junction and thus increase the evidence supporting the junction. The size of these tiles depends on the read size as well as the amount of overlap across the two joined tiles required by the user. For example, for reads that are 36 nt long and a required overlap of at least 10 nucleotides, each fusion junction element is 52 nt long, that is, each tile is 26 nt long. This ensures that every 36-nt read, if mapped to this junction element, will have at least 10 nucleotides mapped to the tile of each gene.

To select the true junction sequence, we determine which fusion junction obtains the highest support, that is, the junction with the highest number of reads aligned to. In addition, we also require the set of single-end reads to be uniformly distributed across the junction to provide further evidence. Provided there is enough coverage overall, we employ a Kolmogorov-Smirnov statistical test, otherwise we apply a simple heuristic by requiring that at least *n *reads align to the junction with at least *m *different starting positions on the junction sequence. The latter parameter ensures that no PCR artifacts affect the junction identification. Also, we search for similarity of the identified junction elsewhere in the genome using BLAT [50], in order to eliminate potential spurious junctions.

From a computational viewpoint, let us assume that we have about 1,000 virtual tiles for each gene. By creating all pair-wise combinations of these virtual tiles for the two genes and considering both directions - gene A upstream of gene B and *vice versa *- will result in 1,000 × 1,000 × 2 = 2 × 10^6 ^putative junctions. If we have approximately 30 candidate fusion transcripts, the putative fusion junction library will thus contain approximately 6 × 10^7 ^= 60 million elements. Using fast alignment tools, this analysis is feasible, although it requires large-scale computational resources. Indeed, we use bowtie to first create an index of the fusion library and then map the reads against it [49]. To fully exploit the parallelization of a multi-node computing cluster, each fusion candidate is analyzed independently on different nodes. Moreover, the fusion junction library itself is also split across multiple nodes in order to optimize the generation of the indexes.

### Sequencing depth and detection of fusion candidates

To assess the impact of sequencing depth on the detection of the fusion candidates, we randomly selected a fraction of mapped reads from sample 2621_D. Specifically, we extracted 10%, 25%, 50%, 75%, and 90% of all PE mapped reads (1.1 million, 3 million, 6 million, 9 million, and 10.8 million PE reads, respectively). The number of fusion candidates with more than five PE reads clearly correlates with sample size: 0, 1, 3, 4, and 7, respectively. The *SLC45A3-ERG *fusion was detected as the top candidate, starting with 3 million mapped PE reads, with a *SPER *of 4.7. The relatively low *SPER *for this candidate is related to the smaller fragment size that has been adopted for this sample (200 nucleotides compared to 300 to 330 nucleotides for the others). The smaller fragment size limits the number of PE reads that could span the junction. From this analysis, it appears that 3 million reads are sufficient for detecting this fusion in this context. However, this result is difficult to generalize. It might be true only for fusion transcripts that are expressed at a similar level to *SLC45A3-ERG*. We cannot exclude the presence of less abundant fusion transcripts that would have been uncovered by deeper sequencing.

### Scoring the candidates

We may take into account different types of information to score the candidates. Potentially we could use the number of PE reads bridging the two genes, the number of reads supporting the main junction, and the 'shape' of the coverage as indicators of the reliability of the candidate. Practically, since it may be possible that the true junction is not detected because of lack of coverage, the more general quantities are based on the number of PE reads supporting the fusion candidate. Hence, every fusion transcript candidate is first scored using *SPER*, the normalized number of supportive PE reads, the most intuitive quantitative measure (see Results - Scoring the candidates). One may argue that a 'local' score - a score that takes into account the expression of the genes involved in the fusion - might be a reasonable choice. We defined *LSPER *(local *SPER*) as the number of inter-transcript PE reads supporting the fusion divided by the average gene expression value computed as RPKM [3]. However, in many cases, only one allele contributes to the fusion transcript. Hence, the expression of the fusion transcript (estimated by the number of inter-transcript reads because the structure of the whole fusion is unknown) may not correlate with the expression of the genes generating it and thus this may impair the correct ranking of the candidates (text and Figure S6 in Additional file 1). After computing *SPER *for each candidate, we need to assign a 'confidence' to this number. We compare it with two expectations. The first one, *DASPER *(the difference between the observed and analytically calculated expected *SPER*), is based on the observation that if two ends were randomly joined, the probability that this occurs for gene A and gene B is proportional to the product of the probability that the two single-ends of the pair are mapped to gene A and gene B:

P(A∩B)=P(A)∗P(B)

where *P*(*A*) and *P*(*B*) are the probabilities that a single-end is mapped to gene A and B, respectively. Note that this is a very conservative estimate because it does not take into account that single ends should also be within a certain distance, based on the fragment size, to be joined in a pair. Nevertheless, as a first approximation, the expected *SPER *can be estimated as the ratio of the number of single-end reads mapped to gene A and gene B and the total number of mapped single-end reads. For the *i*-th candidate, involving gene A and B, we have:

$$\langle SPER_{i}\rangle =\frac{\langle m_{AB}\rangle }{N_{mapped}}\cdot {\text{10}}^{\text{6}}=\frac{\text{1}}{N_{mapped}}\cdot \left\{N_{mapped}\cdot P\left(A\right)\cdot P\left(B\right)\right\}\cdot {\text{10}}^{\text{6}}=\frac{m_{A}\cdot m_{B}}{N_{mapped}^{\text{2}}}\cdot {\text{10}}^{\text{6}}$$

where <*m_AB_*> is the expected number of inter-transcript PE reads under the null hypothesis, and *m_A _*and *m_B _*are the number of single end reads mapped to gene A and B, respectively. By subtracting this number from the observed *SPER*, we can rank the fusion candidates according to *DASPER *score:

$$DASPER_{i}=SPER_{i}-\langle SPER_{i}\rangle$$

We chose to compute the difference between these two quantities compared to a more traditional ratio or log-ratio because it is more robust in cases of low coverage (that is, low number of reads) than computing a ratio. More accurate estimations of the expected *SPER *can certainly be devised for cases with low coverage, although they would likely require the specific characteristics of the sequencing platform and the mapping approach adopted to be taken into account, thus reducing the broader applicability of this method. Although *DASPER *can reliably rank the candidates within a sample, it may be possible that when comparing candidates from multiple samples *DASPER *may not properly account for different fragment sizes. Indeed, smaller fragment sizes decrease the likelihood of sequencing PE reads bridging two genes, resulting in lower *SPER*, and consequently, lower *DASPER*, affecting the comparison among samples. To address this issue, for each fusion transcript candidate *i*, we compute the ratio of its *SPER_i _*to the average *SPER *of all candidates of a sample, that is, *RESPER*:

$$RESPER_{i}=\frac{SPER_{i}}{\frac{\text{1}}{M}\cdot \sum_{j=\text{1}..M}SPER_{j}}$$

where *M *is the total number of fusion transcript candidates for a sample. Since this quantity is independent of the fragment size, it is more suitable for comparisons across samples. Also, as long as the sequencing depth increases, *RESPER *is expected to increase for a real fusion transcript compared to an artifactual one (Figure 3b).

In the case of sufficient coverage, we can also integrate the information related to the junction-sequence identifier analysis, such as the number of single-end reads supporting a junction as well as how evenly the single-end reads cover it. Ideally, the entire fusion junction should be uniformly covered by the reads. If this does not occur, the chimeric transcript might have been generated during sample preparation and the PCR amplification step resulted in an over-representation of that transcript. However, definitive determination of uniform coverage requires great sequencing depth.

### Computational complexity

One of the main issues to address is the computational complexity of processing RNA-Seq data. Computationally, the three modules have different requirements. The fusion transcript detection module depends on the total number of mapped reads. Once the alignment is performed, it takes about 15 minutes to run this module on 20 million mapped PE reads using one core of a dual 2 Intel^® ^Xeon^® ^CPU E5410 at 2.33 GHz (four cores each, for a total of eight cores), with 6 MB cache, 32 GB RAM, and a 156 GB local disk. The filtration cascade module takes about 15 to 30 minutes to run on the same architecture. The difference depends on the number of candidates initially identified. A more intensive effort is required for the junction-sequence identifier analysis, the main bottleneck being the indexing of all the virtual tiles. The time complexity also depends on the size of the region being tiled. The alignment of the reads after the indexing is much less computationally demanding. In fact, the time to complete a junction-sequence identifier analysis for a single candidate in both directions, AB and BA, ranges from about 90 to 180 minutes if run on a single machine. However, by splitting the fusion junction library in different files, it is possible to run the indexing step in parallel, thus substantially decreasing the time complexity. Indeed, by splitting the fusion junction library in files with 2 million elements, it is possible to complete the indexing and the mapping in about 15 minutes for both orientations.

### Report of the analysis results

We also developed a set of tools to report the analysis results through a web interface and the UCSC Genome Browser (text and Figure S7 in Additional file 1) [53]. All programs of FusionSeq take as input one of the standard formats we defined (Additional file 1), and additional tools convert them into files that can be interpreted by the UCSC Genome Browser, such as WIGGLE, BED or GFF. This integration is facilitated by the use of a web interface to interrogate the samples. The user selects the sample and the list of potential candidates is shown with the candidates sorted according to *DASPER *(Figure S7a in Additional file 1). Information regarding the genes involved, such as gene symbols (including aliases), gene description and genomic coordinates are also reported (Figure S7b in Additional file 1). By clicking on the genomic coordinates the corresponding UCSC Genome Browser page is displayed. Also, each candidate has a detailed page reporting detailed information, including the junction-sequence identifier analysis results (Figure S7c in Additional file 1).

Although we extensively rely on the data format of the UCSC Genome Browser, it is not possible to show the results for inter-chromosomal fusions (that is, those between genes on different chromosomes) since it can display only one chromosome at a time. In order to address this issue, we developed SeqViz (Figure S8 in Additional file 1), an application that is based on Circos, an open source software that is particularly suited to the display of genomic information by representing the full genomes as a circle [54]. An example of a Circos image can be found in Figure 4b. Among the main features of Circos is the high flexibility in adding and showing many types of information: connection between the two ends of a PE read, gene annotation sets, expression values, and so on.

### Software and data availability

FusionSeq is available for download at [34]. Data sets used in this study are available via dbGaP [55] (study accession [phs000311.v1.p1]).

## Abbreviations

*DASPER*: difference between the observed and analytically calculated expected *SPER*; EST: expressed sequence tag; GAII: Illumina Genome Analyzer II; nt: nucleotide; PE: paired-end; RESPER: ratio of empirically computed *SPER*s; RNA-Seq: mRNA sequencing; RPKM: reads per kilobase of exon model per million mapped reads; *SPER*: supportive PE reads; UCSC: University of California Santa Cruz.

## Authors' contributions

ASB conceived, developed and implemented FusionSeq, analyzed the data and drafted the manuscript; LH devised, developed and implemented modules of FusionSeq; DP and ST prepared the samples for sequencing, provided feedback for the development of FusionSeq, and experimentally validated the results; DZC developed SeqViz; JSR conceived the use of the abnormal insert-size concept; AKT collected and provided high-quality prostate cancer samples; NK and BJM prepared and sequenced the samples at Rubin lab; MSC sequenced samples at Prognosys Biosciences and provided useful feedback for the development of FusionSeq; FD analyzed the data and drafted the manuscript; MAR coordinated the collection of the samples, provided insights during the development of FusionSeq and the experimental validations, and drafted the manuscript; MBG coordinated the development of FusionSeq, devised some of the filters, helped analyze the data and drafted the manuscript.

## Supplementary Material

Additional file 1 **Supplementary material, tables and figures**. The results of different mapping tools and approaches, the description of additional filters that are annotation specific, more details about data formats, and the visualization tools. Click here for file
