# Supplementary material for: FusionSeq: a modular framework for finding gene fusions by analyzing paired-end RNA-sequencing data
Source: Genome Biol. 2010 Oct 21;11(10):R104. doi: 10.1186/gb-2010-11-10-r104 (PMC3218660; doi:10.1186/gb-2010-11-10-r104)
Supplement: Additional file 1 — Supplementary material, tables and figures. The results of different mapping tools and approaches, the description of additional filters that are annotation specific, more details about data formats, and the visualization tools. [file gb-2010-11-10-r104-S1.PDF]

Supplementary material of:

FusionSeq: a modular framework for finding gene fusions by analyzing Paired-End RNA-Sequencing data

Andrea Sboner[1,2*], Lukas Habegger[1*], Dorothee Pflueger[3], Stephane Terry[3], David Z. Chen[1], Joel S. Rozowsky[2], Ashutosh K. Tewari[4], Naoki Kitabayashi[3], Benjamin J. Moss[3], Mark S. Chee[5], Francesca Demichelis[3,6], Mark A. Rubin[3,*], Mark B. Gerstein[1,2,7,*]

[1] Program in Computational Biology and Bioinformatics, Yale University, 300 George Street, New Haven, CT 06511, USA

[2] Molecular Biophysics and Biochemistry Department, Yale University, 260 Whitney Avenue, New Haven, CT 06520, USA

[3] Department of Pathology & Laboratory Medicine, Weill Cornell Medical College, 1300 York Avenue, New York, NY 10065, USA

[4] Department of Urology, Weill Cornell Medical College, 525 East 68th Street, New York, NY, 10065, USA

[5] Prognosys Biosciences, Inc., 505 Coast Blvd. South, La Jolla, CA 92037, USA

[6] Institute for Computational Biomedicine, Weill Cornell Medical College, 1305 York Avenue, New York, NY 10065, USA

[7] Department of Computer Science, Yale University, 51 Prospect Street, New Haven, CT 06511, USA

* These authors contributed equally to this paper.

Alignment strategy

We employed ELAND v 1.4, included in the Illumina Genome Analyzer II, to map the paired-end reads to the human reference genome (hg18), allowing up to two mismatches. The reference genome was obtained from the UCSC download site (http://hgdownload.cse.ucsc.edu/downloads.html#human). The output of the mapping is included in two “export” files, one for each end. Moreover, we also employed bowtie to map the reads to the genome [49]. However, since bowtie does not allow PE reads to be mapped to two different chromosomes, we mapped the single ends independently, by allowing 2 mismatches and requiring the best alignment to be reported if the read maps to more than one location but less than 11. To explore the issue of multi-mapping we also run bowtie by keeping only the reads that have a unique alignment.

In addition to this analysis, we also aligned the PE reads against a splice junction and a ribosomal library. The splice junction library was generated from UCSC knownGene annotation set by connecting all exons of each transcript pair-wise. We require a minimum of 6bp overlap across the junction for each single end read by creating splice junctions that are 12bp shorter than the twice the read length. In other words, if the read is 36bp the splice junction is 30bp+30bp=60bp.

Concerning the ribosomal library, we used Biomart to download the sequences of all ribosomal genes from Ensembl by selecting rRNA and ribosomal proteins (Gene Ontology term: GO:0005840)[57]. Both for ELAND and bowtie, we aligned the reads simultaneously to all libraries, so that the best alignment is selected.

Comparison of different alignment tools

The results of the alignment with bowtie are reported in **Additional file 1, Table S2** whereas the list of fusion candidates is reported in **Additional file 1, Table S3**. Overall, the number of mapped reads is comparable to that of ELAND with some exceptions (see **Table 1** for comparison). The two samples with 36bp read have a greater number of mapped reads with bowtie. Concerning the difference between the two mapping strategies, the initial unfiltered set of candidates is quite different, with bowtie identifying many more candidates: 18075 fusion candidates (compared to 7342 of ELAND), with an average number of candidates per sample of 2259.4 (range: [1054,3757]). However, the final, filtered, set is comparable including 200 candidates (average number of candidates per sample: 25.0 range: [6-51] – see **Additional file 1, Table S3**), a reduction of 98.9%. Seventy-one (71) bowtie candidates are also found with ELAND (hypergeometric test p-value = $5.9*10^{-78}$). Moreover, 19 out of

the top 20 candidates identified with ELAND are also detected with bowtie. This result suggests that the filters we developed are robust and sufficiently flexible to address different issues of the RNA-Seq experimental procedure, independently from the mapping strategy adopted. It is worth noting that even if we did not optimize the use of bowtie (more efficient and effective ways are certainly possible), the major result is not affected. This sub-optimal use of bowtie is reflected in some candidates that are specific to this tool. It appears that this affects mainly samples with 36bp reads, whereas samples with longer read lengths are less affected.

Comparison of the results when using a canonical splice junction and ribosomal library for mapping the reads

As for the comparison of the results with the ribosomal and splice junction library in the mapping strategy, **Additional file 1, Table S4** shows the results of the mapping, whereas **Additional file 1, Table S5** reports the final list of candidates. The initial list of fusion candidates includes 2272 chimeric transcripts. After the application of the filters, the final set of candidates comprises 136 fusion events, a reduction of 94%. By comparing this list to the one obtained aligning the reads to the genome with ELAND, we find 98 candidates in common (hypergeometric test p-value = $1.0*10^{-110}$). This again demonstrates that our approach is robust to the use of different mapping strategies.

It is also worth noting that the known fusions (*TMPRSS2-ERG, SLC45A3-ERG, NDRG1-ERG*) are always detected by all approaches as well as the novel fusions (*PIGU-ALG5* and *ERG-GMPR*).

Modularity of FusionSeq

The relative independence from the mapping strategy described above is also complemented by the modularity of the framework. This modularity enables the final user the flexibility of assembling the desired analysis pipeline. Indeed, the software programs are independent among each other, sharing only common data formats. As an example, each filter has been developed as a separate software program, thus allowing the user to adapt and optimize the analysis pipeline for the discovery of fusion transcripts. Moreover, although this framework has been developed in C, this feature allows the developers to use different programming languages for the development of their tools. Moreover, to illustrate the use of the different sources of information, **Additional File 1, Figure S9** shows a dependency diagram between the modules and the data sets employed.

Additional filters

FusionSeq implements a few additional filters that are specific to the annotation or the sequencing platform. The main rationale of these filters is to address specific issues and inconsistencies in the annotation itself. Hereafter are the details.

Annotation consistency filter

In order to ensure that the gene annotation set includes consistent information, we developed an annotation consistency filter that removes some clear mis-annotations. For examples, although pseudogenes are not included in the annotation set, a small fraction is present (239) and labeled as such. Also, in an effort to be as consistent as possible, we also remove genes labeled as ribosomal. This filter thus addresses the issue of the annotation description by removing candidates involving gene with a specific user-defined annotation, e.g. ribosomal or pseudogenes.

Proximity filter

Some candidates show PE reads connecting to nearby genes similarly to read-through events. However, the two genes are located on opposite strands. We called these cases *cis* events. When the two genes are located very closely on the genome, a more likely explanation is a mis-annotation of the untranslated region of one gene, probably indicating an extension of that region. Hence, *cis* candidates that are separated by less than *N* (default N=1000) nucleotides are removed.

Blacklist filter

Some fusion candidates are caused by mis-annotations in the relationship between transcripts and the “corresponding” gene. A clear example is the candidate formed by “Human MEN1 region clone epsilon/beta mRNA, 3' fragment (UCSC id = uc001odw.1)” and “Human MEN1 region clone epsilon/beta mRNA, 5' fragment (UCSC id = uc001odv.1)”. The annotation does not consider these as part of the same genes, and thus FusionSeq identifies those as potential candidates. This filter then removes all fusion candidates that have been included in the “blacklist”. The blacklist is user- and application specific.

Splice-junction filter

In order to keep FusionSeq independent as much as possible from the alignment strategy, we do not require that the reads are aligned to a splice-junction library. However, this may lead to some spurious

candidates when a genomic sequence may resemble a junction between two exons. This may be particularly true for processed pseudogenes, where the introns of the parent genes are spliced out and retro-transcribed in the genome. This filter thus maps the reads of a candidate fusion transcript to a splice-junction library (in our case it is based on the UCSC knownGenes). If more than a user-specified fraction of the inter-transcript reads can be mapped to a splice-junction (default 5%) the candidate is discarded. If the original mapping included a splice junction library, we do not expect this filter to have much of an effect.

Mitochondrial filter

If the reference sequence to which the reads are aligned to includes mitochondrial DNA, some of the reads may map to this region. Thus, this filter removes the candidates involving reads aligned to the mitochondrial DNA.

Effects of the filters

The application of the filters reduces the number of fusion transcript candidates by identifying those candidates that are likely to be artifacts generated by the various sources of noise (see **Results – Module #2: PE-read filtration cascade**).

In the case of the alignment against the genome performed using ELAND, we initially had 7342 candidates. After the application of all the filters, we excluded 98% of them, resulting in 133 candidates. To assess if the candidates we removed were actual artifacts, we tested experimentally some of them. **Additional file 1, Table S6** reports the result of the validation, which supports that the excluded candidates are true artifacts. Also, some candidates with negative DASPER were negatively validated, further indicating that they were artifacts likely due to random pairing during sample preparation.

Novel candidates

We were able to identify two novel candidates in the set of samples we used for calibration. The first one is a new fusion involving *ERG* with another partner: the guanosine monophosphate reductase (*GMPR*). In this case, it appears to represent a balanced translocation, since in this sample *ERG* is also rearranged with *TMPRSS2*. Whereas in the *TMPRSS2-ERG* fusions the 3' exons of *ERG* are involved, with *GMPR* the 5' exons 1, 2, and 3 of ERG are included in the fusion. A second candidate include *PIGU*: phosphatidylinositol glycan anchor biosynthesis, class U and *ALG5*: asparagine-linked glycosylation 5, dolichyl-phosphate beta-glucosyltransferase homolog (S. cerevisiae). **Additional file**

1, Figure S2 shows the results of the experimental validations for ERG-GMPR; whereas *PIGU-ALG5* is fully described in Pflueger et al. [43].

Different experimental protocol

An updated experimental protocol was developed at Sanger to improve sequencing with the Illumina Genome Analyzer platform [38]. Among the various steps, they suggest to perform a second round of size selection to reduce the problem of ligation of random cDNA molecule due to inefficient A-tailing. Hence, we resequenced the GM12878 cell line following this protocol. Although we find a reduction in the number of fusion candidates, this reduction does not seem to be as large as one would have expected: 736 and 838 candidates for two lanes, respectively (unfiltered – modified Quail et al. protocol) compared to 1022 (unfiltered – original Illumina protocol).

Simulation results

To further assess the performance of FusionSeq, we performed a simulation study. We employed the GM12878 cell line as an estimate of the background because it is not expected to harbor any fusion transcripts. We randomly generated inter-transcript reads thus simulating the presence of fusion transcripts and added these PE reads to the pool of the actual PE reads of the GM12878 cell line data. We randomly selected 20 gene pairs, by considering their expression levels in this cell line. We assigned each gene pair to different categories depending on which expression quantile bin it falls (we used 4 bins: 50th-60th, 60th-70th, 70th-80th, 80th-90th quantile). We chose 5 gene pairs for each category to have a representation across the dynamic range of gene expression. We randomly selected one exon for each gene and then generated PE reads connecting the two exons. The number of reads generated per fusion depends on the expression of the genes involved in the fusion. From the RPKM values of the genes, we computed the number of reads that may connect the two exons. Moreover, we multiplied this number by a factor F. This factor allows to account for the diploid nature of the human genome, by partially decoupling the expression of the fusion transcript from that of its genes to simulate the real case scenario where, typically, only one allele contributes to a fusion transcript. We then ran FusionSeq with the same parameters as for the original analysis of the cell line and used the scoring methods to compute the sensitivity and the Area Under the ROC (AUC). A DASPER score greater than 1 achieves high sensitivity (0.80) even if the fusion transcript is expressed at half the “wild-type” allele (F=0.5) (see **Additional file 1, Figure S5A**). When the number of inter-transcript PE reads is greated than a quarter (F=0.25) of the expected number (based on the expression of the

genes), the Area Under the ROC curve (AUC) is higher than 0.95 (**Additional file 1, Figure S5B**) demonstrating that the ranking by DASPER enriches for high-confidence fusion transcripts.

Data formats

The results of the mapping with ELAND or bowtie was converted into Mapped Read Format (MRF) part of RSEQtools [58], a computational framework to analyze RNA-Seq data, described in [59]. Hereafter, we describe the data formats specific to FusionSeq.

Gene Fusion Report (GFR) format

We designed a standard format for the list of fusion candidates called Gene Fusion Report (GFR) The rationale is that different filters can be applied to exclude “false positives” artificial fusions starting from an initial set. We also provide a parser that interprets this format allowing the user to propagate easily any changes. For a given fusion candidate, involving gene A and gene B, the basic GFR format requires the following fields:

1. ID: the ID of the fusion candidate: typically it contains the sample name and a unique number separated by an underscore. The number is padded with zeros for consistency;
2. SPER, DASPER and RESPER: see main text for their definition;
3. Number of inter-transcript reads, i.e. the number of pairs having the ends mapped to the two genes;
4. P-value of the insert size distribution analysis for the fusion transcript. Since we do not know the actual composition of the fusion transcript, we computed the p-value for both directions: AB (where gene A is upstream of gene B) and BA (where gene B is upstream of gene A);
5. Number of intra-transcript reads for gene A and gene B, respectively, i.e the number of pairs where both ends map to the same gene;
6. The type of the fusion: inter, intra, read-through and *cis* – see main text **Classifying the candidates**;
7. Name(s) of the transcripts: all the UCSC gene IDs of the isoforms of each gene in the annotation separated by the pipe symbol '|';
8. Chromosome of the genes;
9. Strand information;
10. Start and end coordinates of the longest transcript for both genes;
11. Number of exons in the composite model for both genes;

12. Coordinates of the exons in the composite model: each exon is separated by the pipe symbol '|' and start and end coordinates are comma-separated;
13. Exon-pair count: it describes which exons are connected and the number of inter-reads;
14. Inter-reads: the exon and the coordinates of the reads that join the two genes. Exon number, start and end coordinates are reported as comma-separated, with the pipe symbol '|' separating the different pairs;
15. Reads of the transcripts: the actual sequence of all the inter-reads.

The GFR format can include additional optional information computed in the subsequent processing. For example, it is possible to add gene symbols and descriptions from the UCSC knownGene annotation set.

Breakpoint data format (BP)

Similarly to GFR, the junction-sequence identifier defines a standard format to capture the results of this analysis: BreakPoint data format (BP). For each tile that has at least 1 read aligned to, it reports, comma-separated:

1. chromosome, start and end coordinates of the first tile, using UCSC notation: “chr:start-end”, although the intervals are 1-based and closed;
2. chromosome, start and end coordinates of the second tile
3. All the sequences of the reads mapped to that tile with the offset information, separated by the pipe symbol.

For example, one line may read as:

```
chr21:38764851-38764892,chr21:41758661-41758702,31:GTAGAATCATTCATTTCATTCTTGCAAACCAGCCTGCTTGGCCAGGAGGCA|
30:TGTAGAATCATTCATTTCATTCTTGCAAACCAGCCTGCTTGGCCAGGAGGC
```

where two reads support that specific junction.

Integration with the UCSC Genome Browser and Circos

A number of additional programs have also been developed to display the results of the analysis with the UCSC Genome Browser [53]. All programs can provide as output one of the files that the Genome Browser can interpret.

All the PE reads of a fusion candidate are represented by means of two BED files, one per gene candidate, thus reporting the location of each single read. For candidates that are on the same chromosome, it is possible to create GFF files, showing the connection between the reads (**Additional file 1, Figure S7**). The UCSC Genome Browser, however, cannot represent PE reads that connect genes on different chromosomes.

In order to address this issue we employ Circos, a visualization tool particularly suited for this purpose. [48]. Circos can display various types of data in a circular format. For examples, it can depict all chromosomes on a circle and then draw lines between specific locations, thus enabling the visualization of PE reads connecting different chromosomes. Although Circos is highly flexible and allows the user to display several types of data in a rich way, it pays this flexibility in the level of customizations that are required. We thus developed a web-interface wrapper that simplifies the customization. The user can easily zoom in or out of specific regions through sliding bars and overlay other data on top of the PE reads (**Additional file 1, Figure S8**).

Furthermore, the junction-sequence identification analysis can also be represented by “wiggle” files (WIG), reporting the frequency of the breakpoints for a particular fusion candidate. For each orientation, AB or BA, two wiggle files are generated, one for each gene involved in the fusion. The results of this analysis can also be visualized through the UCSC Genome Browser by means of wiggle files that are generated from all the virtual junctions that have reads supporting them. The wiggle files represent the number of single-end reads supporting the junction, i.e. reads bridging the two genes.

Finally, an intensity file is generated by considering all mapped reads. The file format is that of a bedGraph such that it can be displayed within the UCSC Genome Browser. For each candidate, we can thus have many different tracks that describe the fusion.

Table S1: Fusion candidates for all samples sorted by RESPER. No filtering on DASPER was performed.

ID	Type	Gene 1	Gene 2	SPER	DASPER	RESPER
580_B	intra	ERG	TMPRSS2	36.54	36.53	14.31
1700_D	intra	ERG	TMPRSS2	19.66	19.63	8.79
106_T	intra	ERG	TMPRSS2	10.16	10.11	3.97
2621_D	inter	SLC45A3	ERG	4.29	4.15	3.56
NCIH660	read-through	PSMB8	TAP1	5.58	5.58	2.06
NCIH660	read-through	RNPEP	ELF3	5.58	5.56	2.06
1700_D	inter	ERG	GMPR	4.59	4.59	2.05
NCIH660	intra	ERG	TMPRSS2	5.34	5.34	1.97
1700_D	read-through	SLC16A8	UNQ9336|BAIAP2L2	4.33	4.33	1.93
106_T	read-through	AK094188	AK311452	4.87	4.87	1.9
NCIH660	cis	RPA3	LOC401307	4.61	4.61	1.7
NCIH660	inter	TRIM28	IGFBP2	4.37	3.06	1.61
1700_D	read-through	ZNF473	FLJ26850	3.54	3.54	1.58
580_B	read-through	ZNF577	ZNF649	4.03	4.03	1.58
1043_D	read-through	ZNF577	ZNF649	5.79	5.79	1.55
NCIH660	intra	FOXP1|hFKHLB	RYBP	4.13	4.13	1.52
1700_D	read-through	CAMTA2|KIAA0909	INCA1	3.01	3.01	1.35
NCIH660	read-through	HMGN3	BC070061	3.64	3.62	1.35
1700_D	inter	HDAC5|KIAA0600	EEF1D	2.88	2.84	1.29
1043_D	read-through	FLJ00248	PP14183|LRCH4	4.74	4.74	1.27
99_T	inter	SLC45A3	KLK3	8.58	-57.18	1.2
1700_D	read-through	VMAC	CAPS	2.62	2.62	1.17
NCIH660	cis	PRIM1	HSD17B6	3.16	3.15	1.17
106_T	read-through	FLJ00248	PP14183|LRCH4	2.96	2.96	1.16
1043_D	inter	CR607557	KLK2	4.21	-11.98	1.13
1043_D	cis	AX747861	FLI1	4.21	4.21	1.13
99_T	inter	KLK3	EEF1A1|EEF1A1L14	7.92	-45.53	1.11
NCIH660	read-through	SEC24C	FUT11	2.91	2.9	1.08
NCIH660	inter	HMGN2	EIF5AL1	2.91	2.91	1.08
NCIH660	inter	AK125248	Cap43|NDRG1|AX746885	2.91	2.91	1.08
106_T	read-through	TAGLN	AK126420	2.75	2.75	1.07
580_B	inter	ALG5	AK095356|PIGU	2.73	2.73	1.07
99_T	inter	ERG	Cap43|NDRG1|AX746885	7.26	7.15	1.02
580_B	read-through	ZNF473	FLJ26850	2.47	2.47	0.97
1700_D	read-through	SPAG7	CAMTA2|KIAA0909	2.1	2.1	0.94
99_T	inter	EEF1G	KLK2	6.6	-6.44	0.92
99_T	inter	KLK2	NPY	6.6	-37.16	0.92
106_T	read-through	CEACAM20	AK123850	2.33	2.33	0.91

ID	Type	Gene 1	Gene 2	SPER	DASPER	RESPER
1700_D	read-through	SCO2	TYMP	1.97	1.96	0.88
1700_D	inter	C17orf67	PNPLA7	1.97	1.97	0.88
1043_D	read-through	PPP1R16A	GPT	3.16	3.16	0.84
106_T	read-through	PSMB8	TAP1	2.12	2.11	0.83
106_T	cis	RPA3	LOC401307	2.12	2.12	0.83
1700_D	read-through	UNQ464/PRO809	SYNGR2	1.84	1.83	0.82
NCIH660	read-through	SF3A2	AMH	2.18	2.18	0.81
NCIH660	read-through	NDUFA13	FLJ44968	2.18	2.18	0.81
NCIH660	read-through	CS	CNPY2	2.18	2.17	0.81
NCIH660	cis	BC042096	BC070061	2.18	2.18	0.81
580_B	inter	HIP1R|KIAA0655	CSGALNACT1|ChGn|BC048103	1.95	1.94	0.76
580_B	inter	AK125248	EEF2	1.95	1.94	0.76
580_B	cis	MAK10	GOLM1|GK004	1.95	1.95	0.76
580_B	cis	HSP90B1	DKFZp547P055	1.95	1.95	0.76
1700_D	read-through	FMNL1|AX721309|C17orf1	BC031942|AK311429|AK311564	1.7	1.7	0.76
1700_D	read-through	AK094188	AK311452	1.7	1.7	0.76
1700_D	inter	MAN1C1|pp6318	RHEB	1.7	1.7	0.76
106_T	read-through	VMAC	CAPS	1.91	1.9	0.74
NCIH660	read-through	SCARF2	KLHL22	1.94	1.94	0.72
NCIH660	read-through	GPR19	AK096388	1.94	1.94	0.72
NCIH660	read-through	TNFRSF25	PLEKHG5|KIAA0720	1.94	1.94	0.72
NCIH660	inter	LAMP1	CLDN3	1.94	1.77	0.72
580_B	read-through	FMNL1|AX721309|C17orf1	BC031942|AK311429|AK311564	1.82	1.82	0.71
580_B	read-through	SLC16A8	UNQ9336|BAIAP2L2	1.82	1.82	0.71
106_T	read-through	ZNF577	ZNF649	1.69	1.69	0.66
106_T	read-through	KIF7	BC042063	1.69	1.69	0.66
106_T	read-through	AK023629	CR598484|AJ420566|AK091028	1.69	1.69	0.66
106_T	inter	RP4-695O20__B.10|AK023424	CR614418|MAP3K13	1.69	1.69	0.66
1700_D	read-through	RNPEP	ELF3	1.44	1.44	0.64
NCIH660	read-through	AK057037	AK057037	1.7	1.7	0.63
NCIH660	cis	GRIK5	ZNF574	1.7	1.7	0.63
580_B	inter	TPT1	KLK3	1.56	-49.01	0.61
580_B	read-through	RNPEP	ELF3	1.43	1.42	0.56
580_B	inter	AK125248	TPT1	1.43	1.42	0.56
580_B	inter	AK125248	NR_002819|DKFZp686B0790	1.43	1.43	0.56
NCIH660	inter	AK125248	B2M	1.46	1.46	0.54
NCIH660	inter	VIT	ANAPC13|DKFZp566D193	1.46	1.46	0.54
1700_D	read-through	AK023629	CR598484|AJ420566|AK091028	1.18	1.18	0.53
1700_D	read-through	TIRAP	DCPS	1.18	1.18	0.53
580_B	read-through	HMG1L1	CTCFL|BORIS	1.3	1.3	0.51
580_B	inter	AK125248	NR_002802	1.3	1.3	0.51
106_T	read-through	PAQR6	SMG5	1.27	1.27	0.5

ID	Type	Gene 1	Gene 2	SPER	DASPER	RESPER
106_T	inter	C2orf43	CPNE4	1.27	1.27	0.5
106_T	cis	IL3RA	SLC25A6	1.27	1.27	0.5
106_T	cis	CCDC12	NBEAL2|KIAA0540	1.27	1.27	0.5
2621_D	read-through	SLC16A8	UNQ9336|BAIAP2L2	0.59	0.59	0.49
2621_D	inter	SLC45A3	KLK3	0.59	-61.01	0.49
2621_D	inter	hCPE-R|CLDN4	KLK3	0.59	-96.4	0.49
2621_D	inter	PTMS	KLK3	0.59	-9.4	0.49
1700_D	read-through	PPCS	LOC728621|DKFZp686K01114	1.05	1.05	0.47
1700_D	read-through	ZNF577	ZNF649	1.05	1.05	0.47
1700_D	read-through	ARHGAP27	LOC201175	1.05	1.05	0.47
1700_D	read-through	METRN	AL360260	1.05	1.05	0.47
580_B	read-through	PTPRCAP	CORO1B|DKFZp762I166	1.17	1.17	0.46
580_B	inter	SOX9	EEF2	1.17	-0.77	0.46
580_B	inter	SOCS4	ERG	1.17	1.17	0.46
580_B	cis	TTC19|AL832412	KIAA1047|NCOR1	1.17	1.17	0.46
580_B	cis	TUB	RIC3	1.17	1.17	0.46
1700_D	read-through	KRSP1	KLK4	0.92	0.92	0.41
1700_D	inter	PH-4|DKFZp586D1521	FLJ00108|SPON2	0.92	0.61	0.41
1700_D	inter	ACTG1	EEF2	0.92	-46	0.41
1700_D	inter	CR607557	KLK3	0.92	-5.33	0.41
1700_D	inter	APRG1|C3orf35	KCNQ5	0.92	0.92	0.41
1700_D	cis	LRP2BP	ANKRD37	0.92	0.92	0.41
580_B	read-through	ANKRD23	ANKRD39	1.04	1.04	0.41
580_B	inter	DKFZp761L1918|RHPN2	SLC44A4	1.04	1.03	0.41
580_B	inter	NR_002802	KLK3	1.04	-25.24	0.41
580_B	inter	P4HB	KLK3	1.04	-42.09	0.41
580_B	read-through	AK127124	KIAA1112|ARHGEF4	0.91	0.91	0.36
580_B	read-through	AK094188	AK311452	0.91	0.91	0.36
580_B	inter	P4HB	KLK2	0.91	-22.19	0.36
580_B	inter	AK125248	HNRNPA2B1	0.91	0.91	0.36
580_B	inter	F3	KLK3	0.91	-6.73	0.36
580_B	inter	CR607557	KLK2	0.91	-8.1	0.36
1700_D	read-through	KIAA0819	MICAL3|KIAA1364	0.79	0.79	0.35
1700_D	read-through	C20orf196	CHGB	0.79	0.79	0.35
1700_D	read-through	HRMT1L2|PRMT1	LOC199800	0.79	0.79	0.35
1700_D	read-through	AK055832	TSPAN10|OCSP	0.79	0.79	0.35
1700_D	read-through	TNFSF4	AK127238	0.79	0.79	0.35
1700_D	read-through	CS	CNPY2	0.79	0.78	0.35
1700_D	intra	ARHGDIA|CR601042	FASN|FASN variant protein	0.79	-0.02	0.35
1700_D	inter	SOX9	EEF2	0.79	-1.23	0.35
1700_D	inter	NR4A1	FLJ00108|SPON2	0.79	-0.27	0.35
1700_D	inter	DHCR24	FRAS1|KIAA1500	0.79	0.78	0.35

ID	Type	Gene 1	Gene 2	SPER	DASPER	RESPER
1700_D	inter	HMGN2	EIF5AL1	0.79	0.79	0.35
1700_D	inter	GAPDH|OK/SW-cl.12	KLK3	0.79	-18.13	0.35
1700_D	cis	TUB	RIC3	0.79	0.79	0.35
1700_D	cis	CR627052|AX747178	AX747592	0.79	0.79	0.35
GM12878	inter	HNRNPD	ACTB|ACTG1	0.39	-1.31	0.31
GM12878	inter	GAPDH|OK/SW-cl.12	CALR	0.34	-2.63	0.27
GM12878	read-through	BC110369|AK308561	BC080605	0.29	0.29	0.23
GM12878	inter	LSP1	GAPDH|OK/SW-cl.12	0.29	-1	0.23

*Table S2: Number of mapped PE reads when using bowtie to align the reads against the genome (see **Table 1** for comparison with ELAND).*

Sample ID	Type (PCa=prostate cancer)	Known Fusion Type	Read size	Total Number of PE reads	Mapped PE reads	Percentage of mapped PE reads
106_T	PCa	TMPRSS2-ERG	51	7,239,733	4,031,500	55.69%
1700_D	PCa	TMPRSS2-ERG	51	12,435,299	6,590,255	53.00%
580_B	PCa	TMPRSS2-ERG	36	18,134,550	7,620,978	42.02%
99_T	PCa	NDRG1-ERG	36	2,844,879	1,563,738	54.97%
2621_D	PCa	SLC45A3-ERG	54	22,079,700	11,260,724	51.00%
1043_D	PCa	No known fusions	51	3,003,305	1,450,881	48.31%
NCI-H660	PCa cell line	TMPRSS2-ERG	51	6,512,688	3,572,250	54.85%
GM12878	Lymphoblastoid cell line	No known fusions	54	44,829,991	20,520,958	45.78%

Table S3: Bowtie results. Fusion candidates for all samples sorted by RESPER. No filtering on DASPER was performed. (Sample IDs include a 'b' as a suffix to indicate that the mapping was performed with bowtie)

Sample ID	Type	Gene 1	Gene 2	SPER	DASPER	RESPER
580_Bb	cis	RELA	CCND1	36.48	36.47	13.86
1700_Db	intra	ERG	TMPRSS2	20.18	20.14	10.2
580_Bb	intra	ERG	TMPRSS2	25.46	25.45	9.67
NCIH660b	read-through	cytochrome b	AF079515	17.92	17.56	5.58
106_Tb	intra	ERG	TMPRSS2	11.16	11.1	3.66
NCIH660b	cis	EEF2	SLC25A42	11.48	11.39	3.57
1700_Db	cis	TNRC18|KIAA1856	TSPAN13	5.46	5.46	2.76
580_Bb	read-through	MTND5	cytochrome b	6.17	-4.55	2.34
2621_Db	inter	SLC45A3	ERG	2.66	2.55	2.18
1700_Db	inter	GMPR	ERG	4.25	4.25	2.15
1700_Db	read-through	ZNF473	FLJ26850	4.1	4.1	2.07
106_Tb	intra	STRF6	MTND5	6.2	-3.04	2.03
NCIH660b	intra	ERG	TMPRSS2	5.6	5.6	1.74
106_Tb	read-through	AK094188	AK311452	5.21	5.21	1.71
99_Tb	inter	BC018860	NPY	10.87	-38.98	1.7
580_Bb	read-through	ZNF577	ZNF649	4.07	4.07	1.55
1043_Db	read-through	MTND5	cytochrome b	8.27	-6.14	1.52
99_Tb	inter	cytochrome b	NPY	9.59	-23.59	1.5
1700_Db	read-through	CAMTA2|KIAA0909	INCA1	2.73	2.73	1.38
1700_Db	read-through	SLC16A8	UNQ9336|BAIAP2L2	2.73	2.73	1.38
580_Bb	inter	OK/SW-cl.16	CALR	3.54	-6.6	1.35
GM12878b	read-through	cytochrome b	AF079515	0.63	-0.41	1.32
NCIH660b	read-through	HMGN3	BC070061	4.2	4.18	1.31
1700_Db	inter	OK/SW-cl.16	CALR	2.58	-4.44	1.3
99_Tb	inter	OK/SW-cl.16	CD9	8.31	5.18	1.3
99_Tb	inter	SLC45A3	KLK3	8.31	-47.72	1.3
1700_Db	read-through	VMAC	CAPS	2.43	2.43	1.23
GM12878b	inter	BC018820	EEF1A1	0.58	-1.6	1.22
GM12878b	intra	STRF6	MTND5	0.58	-43.97	1.22
NCIH660b	read-through	PSMB8	TAP1	3.92	3.91	1.22
NCIH660b	inter	OK/SW-cl.16	HNRNPA2B1	3.92	1.71	1.22
NCIH660b	inter	BC018860	UBB	3.92	-7.85	1.22
1043_Db	read-through	ZNF577	ZNF649	6.2	6.2	1.14
NCIH660b	inter	IGFBP2	TRIM28	3.64	2.43	1.13
NCIH660b	inter	OK/SW-cl.16	CR607557	3.64	0.8	1.13
99_Tb	inter	Cap43|NDRG1|AX746885	ERG	7.03	6.94	1.1
99_Tb	inter	BC018820	NPY	7.03	-11.73	1.1

Sample ID	Type	Gene 1	Gene 2	SPER	DASPER	RESPER
2621_Db	inter	OK/SW-cl.16	KLK2	1.33	-86.59	1.09
2621_Db	inter	OK/SW-cl.16	KLK3	1.33	-213.22	1.09
NCIH660b	intra	FOXP1|hFKHLB	RYBP	3.36	3.36	1.05
NCIH660b	read-through	SEC24C	FUT11	3.36	3.35	1.05
2621_Db	inter	cytochrome b	KLK3	1.24	-96	1.02
1043_Db	read-through	FLJ00248	PP14183|LRCH4	5.51	5.51	1.01
1043_Db	inter	cytochrome b	KLK2	5.51	-46.14	1.01
1043_Db	inter	HN1	KLK2	5.51	-74.99	1.01
1043_Db	inter	STRF6	KLK3	5.51	-109.91	1.01
99_Tb	inter	MTND5	NPY	6.39	-10.64	1
1700_Db	cis	NLGN4Y|KIAA0951	NR_001544	1.97	1.97	1
1700_Db	inter	PNPLA7	C17orf67	1.97	1.97	1
1700_Db	inter	OK/SW-cl.16	UBB	1.97	-6.34	1
106_Tb	read-through	FLJ00248	PP14183|LRCH4	2.98	2.98	0.98
106_Tb	read-through	TAGLN	AK126420	2.98	2.97	0.98
NCIH660b	inter	OK/SW-cl.16	NCL	3.08	0.22	0.96
2621_Db	inter	STRF6	KLK3	1.15	-186.83	0.94
1700_Db	read-through	AK094188	AK311452	1.82	1.82	0.92
1700_Db	read-through	SPAG7	CAMTA2|KIAA0909	1.82	1.82	0.92
1700_Db	inter	BC018860	UBB	1.82	-8.12	0.92
1700_Db	cis	BC018820	MTND5	1.82	-11.94	0.92
99_Tb	inter	HN1	P4HB	5.76	-14	0.9
99_Tb	inter	NPY	KLK2	5.76	-32.38	0.9
580_Bb	cis	EEF2	KLK3	2.36	-43.81	0.9
580_Bb	inter	BC018820	KLK2	2.36	-45.79	0.9
1043_Db	inter	LOC644936	CR624170	4.82	4.82	0.88
1043_Db	cis	AX747861	FLI1	4.82	4.82	0.88
1043_Db	inter	MTND5	NR_002802	4.82	1.42	0.88
1043_Db	inter	MTND5	CR607557	4.82	0.06	0.88
1043_Db	inter	MTND5	KLK3	4.82	-33.07	0.88
1043_Db	inter	OK/SW-cl.16	KLK3	4.82	-74.35	0.88
NCIH660b	inter	OK/SW-cl.16	UBB	2.8	-4.04	0.87
NCIH660b	inter	HN1	UBB	2.8	-10.52	0.87
NCIH660b	intra	OK/SW-cl.16	cytochrome b	2.8	-33.73	0.87
2621_Db	inter	MTND5	KLK3	1.07	-95.56	0.87
1700_Db	read-through	FMNL1|AX721309|C17orf1	BC031942|AK311429|AK311564	1.67	1.67	0.84
1700_Db	read-through	SCO2	TYMP	1.67	1.67	0.84
1700_Db	cis	HN1	BC018820	1.67	-43.82	0.84
GM12878b	inter	BC042976|AK098534|JMJD2C|KIAA0780	CR624170	0.39	0.39	0.81
GM12878b	inter	BC018820	C5orf34	0.39	0.38	0.81
106_Tb	intra	OK/SW-cl.16	MTND5	2.48	-6.11	0.81

Sample ID	Type	Gene 1	Gene 2	SPER	DASPER	RESPER
106_Tb	inter	cytochrome b	KLK3	2.48	-28.6	0.81
99_Tb	inter	OK/SW-cl.16	MLPH	5.12	2.8	0.8
580_Bb	inter	BC018820	TPT1	2.1	-8.49	0.8
NCIH660b	cis	BC042096	BC070061	2.52	2.52	0.78
NCIH660b	inter	MTND5	FUS|FUS/ATF-1|TLS/FUS-ERG	2.52	1.1	0.78
NCIH660b	inter	OK/SW-cl.16	B2M	2.52	0.79	0.78
NCIH660b	inter	cytochrome b	HNRNPA2B1	2.52	0.75	0.78
NCIH660b	inter	MTND5	CR607557	2.52	0.5	0.78
NCIH660b	inter	OK/SW-cl.16	ENO1|DKFZp666C036	2.52	-16.06	0.78
NCIH660b	intra	BC018860	STRF6	2.52	-81.46	0.78
1700_Db	cis	FOXP1|hFKHLB	GLT8D4	1.52	1.52	0.77
1700_Db	read-through	cytochrome b	AF079515	1.52	0.98	0.77
580_Bb	cis	TBC1D3F|PRC17|TBC1D3E| TBC1D3H|TBC1D3	NPEPPS	1.97	1.97	0.75
106_Tb	read-through	VMAC	CAPS	2.23	2.23	0.73
106_Tb	read-through	BC018860	OK/SW-cl.16	2.23	-25.86	0.73
2621_Db	intra	OK/SW-cl.16	MTND5	0.89	-26.47	0.73
99_Tb	inter	STRF6	GOLM1|GK004	4.48	-1.74	0.7
99_Tb	inter	OK/SW-cl.16	KLK4	4.48	-8.8	0.7
99_Tb	inter	OK/SW-cl.16	PSAP	4.48	-13.95	0.7
99_Tb	intra	CALR	KLK3	4.48	-28.27	0.7
580_Bb	read-through	ZNF473	FLJ26850	1.84	1.84	0.7
580_Bb	cis	MAK10	GOLM1|GK004	1.84	1.83	0.7
580_Bb	inter	OK/SW-cl.16	CACNA1D	1.84	-1.11	0.7
NCIH660b	cis	RPA3	LOC401307	2.24	2.24	0.7
NCIH660b	cis	MTX1	GBA	2.24	2.24	0.7
NCIH660b	cis	FAM105B	ANKH	2.24	2.24	0.7
NCIH660b	read-through	CS	CNPY2	2.24	2.23	0.7
NCIH660b	inter	OK/SW-cl.16	ATP2C1	2.24	1.22	0.7
NCIH660b	inter	OK/SW-cl.16	FUS|FUS/ATF-1|TLS/FUS-ERG	2.24	0.25	0.7
1700_Db	inter	MAN1C1|pp6318	RHEB	1.37	1.37	0.69
1700_Db	read-through	UNQ464/PRO809	SYNGR2	1.37	1.37	0.69
1700_Db	read-through	RNPEP	ELF3	1.37	1.36	0.69
1700_Db	inter	EEF1D	HDAC5|KIAA0600	1.37	1.33	0.69
1700_Db	inter	OK/SW-cl.16	FUS|FUS/ATF-1|TLS/FUS-ERG	1.37	-0.35	0.69
106_Tb	intra	DKFZp547E087	AK055742	1.98	1.98	0.65
106_Tb	read-through	AK023629	CR598484|AJ420566|AK091028	1.98	1.98	0.65
106_Tb	inter	OK/SW-cl.16	CALR	1.98	-0.72	0.65
580_Bb	read-through	SLC16A8	UNQ9336|BAIAP2L2	1.71	1.71	0.65
1700_Db	read-through	AK023629	CR598484|AJ420566|AK091028	1.21	1.21	0.61
1700_Db	read-through	METRN	AL360260	1.21	1.21	0.61
1700_Db	intra	SMPD4	FAM128A	1.21	1.21	0.61

Sample ID	Type	Gene 1	Gene 2	SPER	DASPER	RESPER
1700_Db	inter	OK/SW-cl.16	SLC25A6	1.21	1.04	0.61
1700_Db	inter	STRF6	CR607557	1.21	-9.35	0.61
1700_Db	inter	OK/SW-cl.16	GAPDH|OK/SW-cl.12	1.21	-17.83	0.61
1700_Db	inter	MTND5	KLK3	1.21	-21.81	0.61
1700_Db	intra	STRF6	cytochrome b	1.21	-84.35	0.61
GM12878b	inter	OK/SW-cl.16	NCL	0.29	-3.41	0.61
NCIH660b	inter	STRF6	BC030591|TAF15|RBP56/CHN	1.96	1.23	0.61
NCIH660b	inter	OK/SW-cl.16	HMGB3|DKFZp779G118	1.96	-0.95	0.61
NCIH660b	inter	OK/SW-cl.16	CALR	1.96	-4.07	0.61
99_Tb	inter	STRF6	SLC45A3	3.84	-21.3	0.6
580_Bb	cis	HSP90B1	DKFZp547P055	1.57	1.57	0.6
580_Bb	inter	OK/SW-cl.16	BC030591|TAF15|RBP56/CHN	1.57	0.8	0.6
580_Bb	inter	cytochrome b	CALR	1.57	-3.2	0.6
580_Bb	inter	STRF6	TPT1	1.57	-23.36	0.6
580_Bb	inter	BC018820	KLK3	1.57	-94.18	0.6
2621_Db	intra	STRF6	MTND5	0.71	-23.26	0.58
106_Tb	read-through	CEACAM20	AK123850	1.74	1.74	0.57
106_Tb	inter	cytochrome b	KLK2	1.74	-18.18	0.57
580_Bb	cis	TBC1D3C|TBC1D3G|PRC17|TBC1D3	NPEPPS	1.44	1.44	0.55
580_Bb	inter	ALG5	AK095356|PIGU	1.44	1.44	0.55
1700_Db	read-through	PPCS	LOC728621|DKFZp686K01114	1.06	1.06	0.54
1700_Db	read-through	TIRAP	DCPS	1.06	1.06	0.54
1700_Db	inter	OK/SW-cl.16	BC030591|TAF15|RBP56/CHN	1.06	0.35	0.54
1700_Db	inter	STRF6	PTMS	1.06	-1.94	0.54
1700_Db	inter	STRF6	MLPH	1.06	-2.47	0.54
1700_Db	inter	OK/SW-cl.16	CR607557	1.06	-6.81	0.54
1700_Db	inter	BC018820	KLK2	1.06	-26.48	0.54
1700_Db	inter	HN1	KLK2	1.06	-49.35	0.54
NCIH660b	read-through	AK094188	AK311452	1.68	1.68	0.52
NCIH660b	read-through	GPR19	AK096388	1.68	1.68	0.52
NCIH660b	inter	VIT	ANAPC13|DKFZp566D193	1.68	1.68	0.52
NCIH660b	read-through	TNFRSF25	PLEKHG5|KIAA0720	1.68	1.68	0.52
NCIH660b	inter	OK/SW-cl.16	HNRNPA3	1.68	1.27	0.52
NCIH660b	inter	OK/SW-cl.16	BC030591|TAF15|RBP56/CHN	1.68	1	0.52
NCIH660b	inter	OK/SW-cl.16	SFTPH	1.68	0.77	0.52
NCIH660b	inter	HN1	NR_002802	1.68	-3.71	0.52
NCIH660b	inter	BC018820	Cap43|NDRG1|AX746885	1.68	-13.56	0.52
2621_Db	inter	BC018860	KLK2	0.62	-89.2	0.51
580_Bb	read-through	FMNL1|AX721309|C17orf1	BC031942|AK311429|AK311564	1.31	1.31	0.5
580_Bb	intra	CALR	KLK3	1.31	-10.96	0.5
580_Bb	intra	BC018860	MTND5	1.31	-11.84	0.5

Sample ID	Type	Gene 1	Gene 2	SPER	DASPER	RESPER
580_Bb	inter	OK/SW-cl.16	TPT1	1.31	-18.7	0.5
106_Tb	read-through	KIF7	BC042063	1.49	1.49	0.49
106_Tb	read-through	PAQR6	SMG5	1.49	1.48	0.49
106_Tb	inter	STRF6	KLK3	1.49	-46.54	0.49
1700_Db	read-through	TNFSF4	AK127238	0.91	0.91	0.46
1700_Db	read-through	HRMT1L2|PRMT1	LOC199800	0.91	0.91	0.46
1700_Db	read-through	KRSP1	KLK4	0.91	0.91	0.46
1700_Db	inter	OK/SW-cl.16	SLC25A6	0.91	0.9	0.46
1700_Db	inter	OK/SW-cl.16	NR_002802	0.91	-2.33	0.46
1700_Db	inter	OK/SW-cl.16	NR4A1	0.91	-2.81	0.46
1700_Db	inter	BC018860	PMEPA1	0.91	-5.06	0.46
1700_Db	inter	BC018820	KLK3	0.91	-40.68	0.46
1700_Db	inter	BC018860	KLK2	0.91	-66.29	0.46
580_Bb	inter	SOCS4	ERG	1.18	1.18	0.45
580_Bb	cis	TTC19|AL832412	KIAA1047|NCOR1	1.18	1.18	0.45
580_Bb	read-through	RNPEP	ELF3	1.18	1.17	0.45
580_Bb	inter	STRF6	LRP5	1.18	-0.18	0.45
580_Bb	inter	BC018820	PSAP	1.18	-2.82	0.45
580_Bb	inter	STRF6	UBB	1.18	-9.13	0.45
580_Bb	cis	TUB	RIC3	1.05	1.05	0.4
580_Bb	inter	SLC44A4	DKFZp761L1918|RHPN2	1.05	1.04	0.4
580_Bb	inter	OK/SW-cl.16	MLPH	1.05	-1.4	0.4
580_Bb	inter	BC018860	TPT1	1.05	-10.55	0.4
580_Bb	inter	STRF6	NR_002819|DKFZp686B0790	1.05	-12.46	0.4
580_Bb	inter	P4HB	KLK3	1.05	-35.87	0.4
580_Bb	read-through	AK094188	AK311452	0.92	0.92	0.35
580_Bb	read-through	AK127124	KIAA1112|ARHGEF4	0.92	0.92	0.35
580_Bb	read-through	ANKRD23	ANKRD39	0.92	0.92	0.35
580_Bb	inter	SOX9	EEF2	0.92	-0.67	0.35
580_Bb	inter	OK/SW-cl.16	HNRNPA2B1	0.92	-1.9	0.35
580_Bb	inter	F3	KLK3	0.92	-5.88	0.35
580_Bb	inter	cytochrome b	P4HB	0.92	-13.45	0.35
580_Bb	inter	BC018860	EEF2	0.92	-21.18	0.35
580_Bb	inter	TPT1	KLK3	0.92	-23.31	0.35
580_Bb	inter	HN1	EEF2	0.92	-32.68	0.35
580_Bb	inter	OK/SW-cl.16	NME4	0.79	-2.44	0.3
580_Bb	inter	STRF6	ABCC4|MOAT-B	0.79	-2.81	0.3
580_Bb	inter	STRF6	HSPA1B|HSPA1A	0.79	-10.26	0.3
580_Bb	inter	P4HB	KLK2	0.79	-17.77	0.3
580_Bb	cis	HN1	BC018820	0.79	-68.9	0.3

*Table S4: Number of mapped PE reads when using ELAND to align the reads against the genome, the splice junction library and a ribosomal library (see **Table 1** for comparison with ELAND).*

**Sample ID**	**Type** (PCa=prostate cancer)	**Known Fusion Type**	**Read size**	**Total Number of PE reads**	**Mapped PE reads**	**Percentage of mapped PE reads**
106_T	PCa	TMPRSS2-ERG	51	7,239,733	4,087,713	56%
1700_D	PCa	TMPRSS2-ERG	51	12,435,299	6,048,743	49%
580_B	PCa	TMPRSS2-ERG	36	18,134,550	6,574,373	36%
99_T	PCa	NDRG1-ERG	36	2,844,879	1,677,113	59%
2621_D	PCa	SLC45A3-ERG	54	22,079,700	12,417,402	56%
1043_D	PCa	No known fusions	51	3,003,305	1,624,692	54%
NCI-H660	PCa cell line	TMPRSS2-ERG	51	6,512,688	3,670,006	56%
GM12878	Lymphoblastoid cell line	No known fusions	54	44,829,991	13,189,364	29%

Table S5: ELAND results when aligning to a splice junction and a ribosomal library. Fusion candidates for all samples sorted by RESPER. No filtering on DASPER was performed.

ID	Type	Gene 1	Gene2	SPER	DASPER	RESPER
580_B	intra	ERG	TMPRSS2	55.97	55.96	16.94
1700_D	intra	ERG	TMPRSS2	21.82	21.75	8.85
2621_D	inter	SLC45A3	ERG	3.95	3.78	4.35
106_T	intra	ERG	TMPRSS2	11.99	11.9	4.13
NCIH660	read-through	HMGN3	BC070061	14.71	14.68	4.01
1700_D	inter	GMPR	ERG	5.79	5.78	2.35
1700_D	read-through	SLC16A8	UNQ9336|BAIAP2L2	5.29	5.29	2.14
580_B	read-through	ZNF577	ZNF649	6.39	6.39	1.93
1700_D	read-through	ZNF473	FLJ26850	4.3	4.3	1.74
NCIH660	read-through	PSMB8	TAP1	6.27	6.26	1.71
NCIH660	read-through	RNPEP	ELF3	6.27	6.23	1.71
99_T	inter	AER61|C3orf64	AK002196|BC034812	7.75	7.75	1.55
99_T	inter	SLC45A3	KLK3	7.75	-59.16	1.55
1700_D	read-through	CAMTA2|KIAA0909	INCA1	3.8	3.8	1.54
106_T	read-through	AK094188	AK311452	4.16	4.16	1.43
1700_D	read-through	VMAC	CAPS	3.47	3.47	1.41
NCIH660	intra	ERG	TMPRSS2	4.9	4.9	1.34
NCIH660	inter	IGFBP2	TRIM28	4.9	2.1	1.34
1043_D	read-through	FLJ00248	PP14183|LRCH4	5.54	5.54	1.23
1043_D	read-through	ZNF577	ZNF649	5.54	5.54	1.23
1700_D	inter	EEF1D	HDAC5|KIAA0600	2.98	2.89	1.21
NCIH660	read-through	SF3A2	AMH	4.36	4.35	1.19
NCIH660	cis	RPA3	LOC401307	4.36	4.36	1.19
106_T	read-through	FLJ00248	PP14183|LRCH4	3.42	3.42	1.18
1700_D	read-through	SPAG7	CAMTA2|KIAA0909	2.81	2.81	1.14
106_T	read-through	CEACAM20	AK123850	3.18	3.18	1.09
106_T	read-through	TAGLN	AK126420	3.18	3.18	1.09
1043_D	inter	CR607557	KLK2	4.92	-18.2	1.09
99_T	inter	PPAP2A|PAP2-a1	NPY	5.37	-0.2	1.07
99_T	inter	TPT1	KLK3	5.37	-27.01	1.07
2621_D	inter	CREB3L1|OASIS	KLK3	0.97	-38.8	1.06
NCIH660	intra	FOXP1|hFKHLB	RYBP	3.81	3.81	1.04
1700_D	read-through	SCO2	TYMP	2.48	2.48	1.01
GM12878	inter	EDNRA|ETA	GTF2F2	0.45	0.45	1
1043_D	cis	AX747861	FLI1	4.31	4.31	0.95
99_T	inter	SERPINB6	KLK3	4.77	-4.4	0.95
99_T	inter	PTMS	KLK3	4.77	-4.39	0.95
1700_D	read-through	LRFN1	BC110060	2.31	2.31	0.94
NCIH660	read-through	SEC24C	FUT11	3.27	3.25	0.89

ID	Type	Gene 1	Gene2	SPER	DASPER	RESPER
NCIH660	inter	VIT	ANAPC13|DKFZp566D193	3.27	3.27	0.89
NCIH660	cis	PRIM1	HSD17B6	3.27	3.27	0.89
2621_D	inter	P4HB	KLK3	0.81	-75.33	0.89
580_B	inter	ALG5	AK095356|PIGU	2.89	2.89	0.87
1700_D	read-through	UNQ464/PRO809	SYNGR2	2.15	2.15	0.87
1700_D	read-through	FMNL1|AX721309|C17orf1	BC031942|AK311429|AK311564	2.15	2.15	0.87
1700_D	inter	PNPLA7	C17orf67	2.15	2.15	0.87
106_T	read-through	PSMB8	TAP1	2.45	2.44	0.84
106_T	read-through	ZNF577	ZNF649	2.45	2.45	0.84
99_T	inter	Cap43|NDRG1|AX746885	ERG	4.17	4.06	0.83
1043_D	read-through	PPP1R16A	GPT	3.69	3.69	0.82
580_B	cis	MAK10	GOLM1|GK004	2.59	2.58	0.78
106_T	read-through	VMAC	CAPS	2.2	2.2	0.76
106_T	read-through	AK023629	CR598484|AJ420566|AK091028	2.2	2.2	0.76
NCIH660	read-through	NDUFA13	FLJ44968	2.72	2.72	0.74
1700_D	inter	MAN1C1|pp6318	RHEB	1.82	1.82	0.74
99_T	intra	SPINT2|kop	KLK3	3.58	-14.44	0.71
99_T	intra	CALR	KLK3	3.58	-47.56	0.71
2621_D	inter	hCPE-R|CLDN4	KLK3	0.64	-116.51	0.71
580_B	read-through	ZNF473	FLJ26850	2.28	2.28	0.69
580_B	cis	HSP90B1	DKFZp547P055	2.28	2.28	0.69
1043_D	read-through	AK023629	CR598484|AJ420566|AK091028	3.08	3.08	0.68
106_T	read-through	KIF7	BC042063	1.96	1.96	0.67
1700_D	read-through	RNPEP	ELF3	1.65	1.65	0.67
1700_D	read-through	GLYCTK1|GLYCTK	DNAH1|KIAA1410	1.65	1.65	0.67
1700_D	read-through	AK094188	AK311452	1.65	1.65	0.67
1700_D	inter	PIAS1	UTX	1.65	1.65	0.67
NCIH660	read-through	CS	CNPY2	2.45	2.43	0.67
NCIH660	read-through	TNFRSF25	PLEKHG5|KIAA0720	2.45	2.45	0.67
NCIH660	cis	BC042096	BC070061	2.45	2.45	0.67
NCIH660	cis	GRIK5	ZNF574	2.45	2.45	0.67
580_B	read-through	FMNL1|AX721309|C17orf1	BC031942|AK311429|AK311564	2.13	2.13	0.64
580_B	read-through	SLC16A8	UNQ9336|BAIAP2L2	2.13	2.13	0.64
2621_D	read-through	SLC16A8	UNQ9336|BAIAP2L2	0.56	0.56	0.62
2621_D	inter	SLC45A3	KLK3	0.56	-79.84	0.62
2621_D	inter	PTMS	KLK3	0.56	-13.48	0.62
2621_D	cis	EEF2	KLK3	0.56	-310.1	0.62
1700_D	inter	APRG1|C3orf35	KCNQ5	1.49	1.49	0.6
99_T	inter	PSAP	KLK3	2.98	-50.53	0.6
NCIH660	read-through	SCARF2	KLHL22	2.18	2.18	0.59
NCIH660	read-through	GPR19	AK096388	2.18	2.18	0.59
106_T	read-through	SCO2	TYMP	1.71	1.71	0.59

ID	Type	Gene 1	Gene2	SPER	DASPER	RESPER
106_T	read-through	PAQR6	SMG5	1.71	1.71	0.59
1700_D	read-through	AK023629	CR598484|AJ420566|AK091028	1.32	1.32	0.54
1700_D	read-through	ZNF577	ZNF649	1.32	1.32	0.54
1700_D	read-through	METRN	AL360260	1.32	1.32	0.54
2621_D	inter	FASN|FASN variant protein	KLK2	0.48	-24.01	0.53
2621_D	inter	MUC6	KLK2	0.48	-7.24	0.53
NCIH660	cis	FAM105B	ANKH	1.91	1.9	0.52
580_B	read-through	RNPEP	ELF3	1.67	1.65	0.51
580_B	cis	TTC19|AL832412	KIAA1047|NCOR1	1.67	1.67	0.51
106_T	read-through	ANKRD23	ANKRD39	1.47	1.47	0.51
106_T	cis	RPA3	LOC401307	1.47	1.47	0.51
106_T	cis	CCDC12	NBEAL2|KIAA0540	1.47	1.47	0.51
106_T	cis	THADA|KIAA1767|GITA/3p fusion|GITA-A2	PRKCE	1.47	1.47	0.51
1700_D	read-through	C20orf196	CHGB	1.16	1.16	0.47
1700_D	read-through	ANKRD23	ANKRD39	1.16	1.16	0.47
1700_D	read-through	TIRAP	DCPS	1.16	1.16	0.47
1700_D	inter	SOX9	EEF2	1.16	-3.13	0.47
1700_D	inter	COL9A2	EWSR1	1.16	1.06	0.47
1700_D	cis	LRP2BP	ANKRD37	1.16	1.16	0.47
580_B	inter	SOX9	EEF2	1.52	-2.42	0.46
580_B	inter	CD63	KLK3	1.52	-14.42	0.46
NCIH660	read-through	JAG2	UGPP|NUDT14	1.63	1.63	0.45
NCIH660	intra	CTNND2	GNPDA1	1.63	1.63	0.45
NCIH660	cis	IL17RB	ACTR8	1.63	1.63	0.45
2621_D	inter	PSAP	KLK3	0.4	-56.3	0.44
580_B	read-through	NR_001544	TTY14	1.37	1.37	0.41
580_B	read-through	ANKRD23	ANKRD39	1.37	1.37	0.41
580_B	read-through	PTPRCAP	CORO1B|DKFZp762I166	1.37	1.37	0.41
580_B	inter	SOCS4	ERG	1.37	1.37	0.41
580_B	cis	TUB	RIC3	1.37	1.37	0.41
1700_D	read-through	KIAA0819	MICAL3|KIAA1364	0.99	0.99	0.4
1700_D	read-through	HRMT1L2|PRMT1	LOC199800	0.99	0.99	0.4
1700_D	read-through	AK055832	TSPAN10|OCSP	0.99	0.99	0.4
1700_D	read-through	C11orf2	TM7SF2	0.99	0.9	0.4
1700_D	read-through	TNFSF4	AK127238	0.99	0.99	0.4
1700_D	inter	FLJ00108|SPON2	EEF2	0.99	-71.42	0.4
1700_D	inter	FLJ00108|SPON2	NR4A1	0.99	-0.99	0.4
1700_D	inter	PH-4|DKFZp586D1521	FLJ00108|SPON2	0.99	0.26	0.4
1700_D	inter	DHCR24	FRAS1|KIAA1500	0.99	0.97	0.4
NCIH660	cis	KIAA1380|JMJD1C|DKFZp761F0118	REEP3	1.36	1.36	0.37
580_B	inter	SLC44A4	DKFZp761L1918|RHPN2	1.22	1.19	0.37

ID	Type	Gene 1	Gene2	SPER	DASPER	RESPER
580_B	inter	NR_002802	KLK3	1.22	-37.28	0.37
580_B	inter	CR607557	KLK3	1.22	-23.44	0.37
580_B	inter	P4HB	KLK3	1.22	-62.39	0.37
580_B	read-through	STX16	NPEPL1	1.06	1.06	0.32
580_B	read-through	AK127124	KIAA1112|ARHGEF4	1.06	1.06	0.32
580_B	read-through	AK094188	AK311452	1.06	1.06	0.32
580_B	inter	P4HB	KLK2	1.06	-32.32	0.32
580_B	inter	F3	KLK3	1.06	-11.37	0.32
580_B	inter	CR607557	KLK2	1.06	-11.88	0.32
580_B	read-through	VMAC	CAPS	0.91	0.91	0.28
580_B	inter	MLC-3|MYL6	KLK3	0.91	-12.79	0.28
580_B	inter	PLA2G2A	KLK3	0.91	-30.89	0.28
580_B	inter	PTMS	KLK3	0.91	-6.31	0.28
580_B	cis	EEF2	KLK2	0.91	-62.22	0.28

Table S6: Experimental validation of some of the candidates that are removed by the filters or "demoted" by DASPER (in red). By negative, we indicate that no amplicon was identified from agarose gel electrophoresis and direct Sanger-sequencing after PCR. The table reports the SPER, DASPER, and RESPER values as well as the filter that removed the candidate.

Candidate	SPER	DASPER	RESPER	Filtered by	Experimental validation
HFM1-BC048201	445.11	445.06	31.97	Ribosomal	Negative
DLG2-UNC45B	4815.35	4809.55	345.83	Ribosomal	Negative
HSP90B1-LRRC28	50.48	50.48	3.63	Small scale sequence similarity	Negative
SOX9-EEF2	1.17	-0.77	0.87	None	Negative
KLK2-P4HB	0.91	-22.19	0.67	None	Negative

Table S7: Known isoforms of TMPRSS2-ERG. The table reports the exons involved in the fusion junction for each isoform. The exon numbers are referred to NCBI GenBank Accession numbers NM_005656.3 (TMPRSS2) and NM_004449.4 (ERG).

Isoforms	TMPRSS2 exons	ERG exons	References
I (TMPRSS2:ERGb)	1	2-13	Wang et al. [46], Tomlins et al. [23]
II	1	3-13	Wang et al. [46]
III (TMPRSS2:ERGa)	1	5-13	Wang et al. [46], Tomlins et al. [23]
IV	1	6b-13	Wang et al. [46]
V	1,3	2-13	Wang et al. [46]
VI	1,3	5-13	Wang et al. [46]
VII	1,3	6b-13	Wang et al. [46]
VIII	1,3,4	5-13	Wang et al. [46]
T2/E3	1,3	3-13	Liu et al. 2007 [60]
T1/E6	1	7-13	Clark et al. 2007 [56]
T4/E4	1,3,4,5	5-13	Clark et al. 2007 [56]
T4/E5	1,3,4,5	6b-13	Clark et al. 2007 [56]
T5/E4	1,3,4,5,6	5-13	Clark et al. 2007 [56]

Supplementary Figure legend

Figure S1: Insert-size analysis. A*. The insert-size computation can help identifying structural variations such as insertions and deletions, by comparing to the normal insert-size distribution. Deletions will result in bigger insert sizes, whereas insertions are characterized by smaller insert sizes.* ***B.*** *The direct application of this principle to the transcriptome is not possible. Three layers of complexity compared to the reference genome can prevent the direct use of the insert-size analysis: 1. germline variations of the individual genome; 2. somatic variations of the cancer genome; and 3. splicing and alternative splicing. If PE reads are mapped to the transcriptome (unknown) the insert-size of normal transcripts will be comparable to the fragment size. However, since PE reads are mapped to the genome, the insert size is not meaningful anymore. PE reads of normal spliced genes may have a bigger insert size (hashed light blue) compared to PE reads of fused genes (hashed blue-yellow). Hashed symbols highlight the correspondence of the reads from the transcriptome to the genome.*

Figure S2: Novel fusion candidate: experimental validation through PCR and Sanger sequencing. *Transcripts of ERG-GMPR fusion were amplified by PCR using forward primer in ERG exon 2 and using reverse primer in GMPR exon 8. The schematic reports the ERG-GMPR fusion transcript along with the Sanger sequencing result representing the breakpoint sequence. Also, a schematic of ERG fusions by translocating 3' sequences to TMPRSS2 gene on chr. 21 and 5' sequences to GMPR gene on chr. 6 is reported.*

Figure S3. Candidate fusion between AX747861 and FLI1*. EST evidence suggests that this cis fusion event, i.e. event between neighboring genes on opposite strands, may in reality be a longer isoform of FLI1. Indeed, the "chimeric" PE reads align to the region where there is EST evidence of a single FLI1 isoform (green box).*

Figure S4. Validation of the minor breakpoint. A. *The location of the breakpoints and the sequences of the junctions as well as a subset of the aligned reads is reported. The minor*

breakpoint between TMPRSS2 exon 1 and ERG exon 6b in sample 1700_D is consistent with the expression of isoform IV. ***B.*** *A PCR assay designed specifically for isoform IV, detects a single 95bp PCR product.*

Figure S5. Simulation results**. **A. *The sensitivity of FusionSeq as a function of the factor F, i.e. the fraction of inter-transcript reads generated to simulate the presence of a fusion transcript.* ***B.*** *ROC curves are reported for the scoring methods SPER and DASPER for different values of the factor F.*

Figure S6. Effects of the different scores for each sample. *The fusion transcript candidates are sorted by DASPER (x-axis), whereas SPER, RESPER and LSPER are plotted for each candidate. LSPER is computed by dividing the number of inter-transcript reads with the sum of the RPKM values of the genes generating the candidate (Mortazavi et al. 2008). In several cases, the LSPER of the top candidate has low values (e.g. 106_T, 2621_D, 1700_D, etc.). This suggests that the genes involved in the fusion are expressed at relatively higher levels than those of other fusion candidates. Hence, this measure would have impaired the proper detection and ranking of the fusion candidates.*

Figure S7. Snapshot of the web-interface of FusionSeq to interrogate the results of the computational framework. A. *Sample name, minimum number of PE reads and type of the fusion candidates can be selected.* ***B.*** *The list of the candidates are reported along with the statistics we introduce with FusionSeq. The hyperlink directly access UCSC GenomeBrowser to display the location of the genes.* ***C.*** *Each candidate has a detailed page with additional details about the fusion, such as the number or inter-transcript reads, the connectivity among exons. If the two genes are located on the same chromosome, a hyperlink points directly to the UCSC Genome Browser and shows all the reads. Moreover, the junction-sequence identifier result can be accessed by clicking on the icons showing the possible combinations, AB or BA. Furthermore, the expression signal of the chromosomes can be loaded as additional track on the Genome Browser.*

Figure S8. Representing the PE reads with Circos. *PE reads connecting genes on different chromosomes can be easily depicted with Circos. Although very flexible, it requires the user to manually specify all the parameters within configuration files. To hide this process to the final user, we developed SeqViz: a user-interface that automatically allows the user to display all the reads in a specified region as well as further magnifying sections of that region. Moreover, it allows to seamlessly overlay additional information such as the gene annotation, the exons of the genes as well as the signal tracks.*

Figure S9. Dependency diagram. *The list of data sources and the modules/filters that use these data is depicted.*

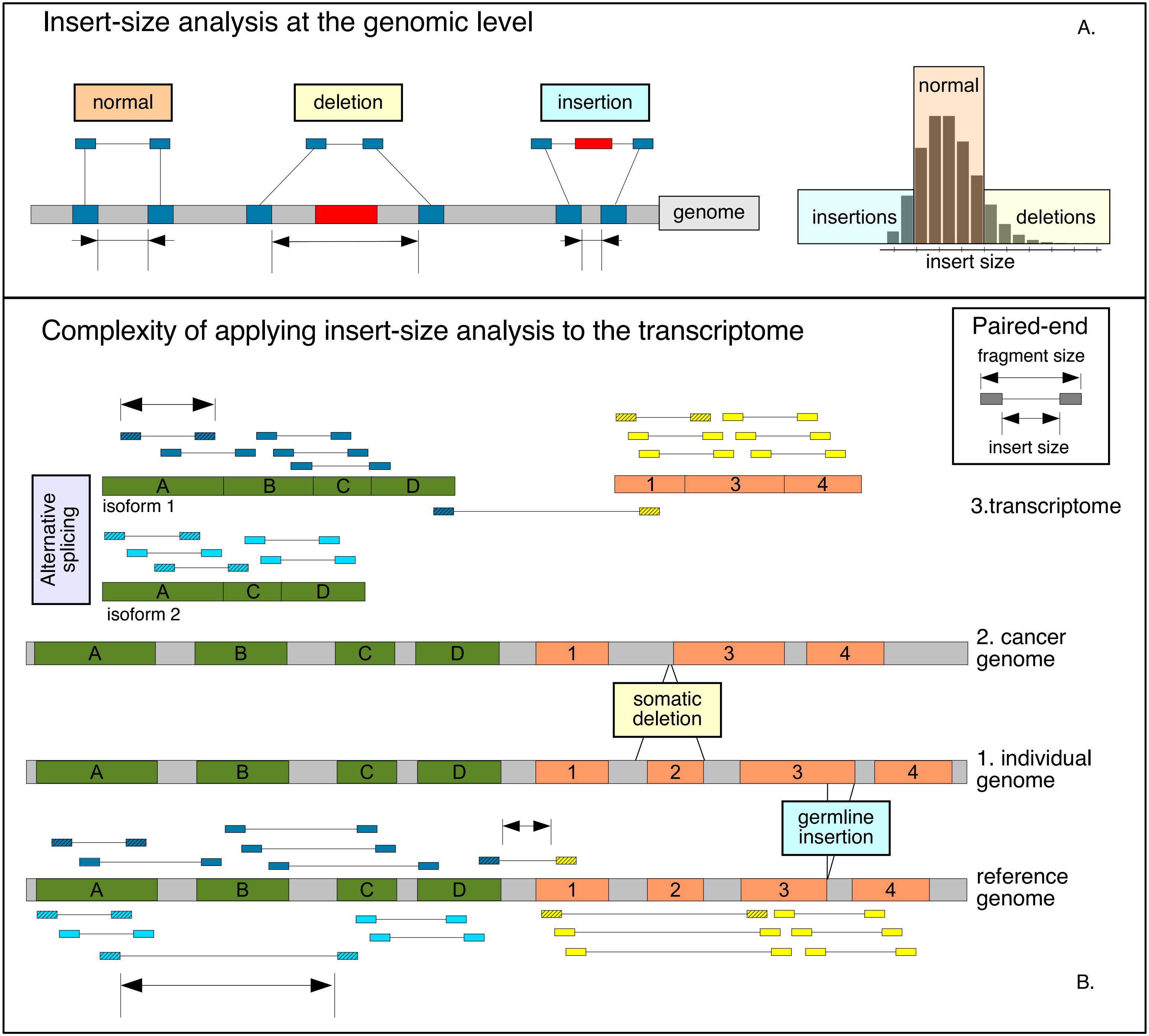

Supplementary Figure 1 Rubin/Gerstein

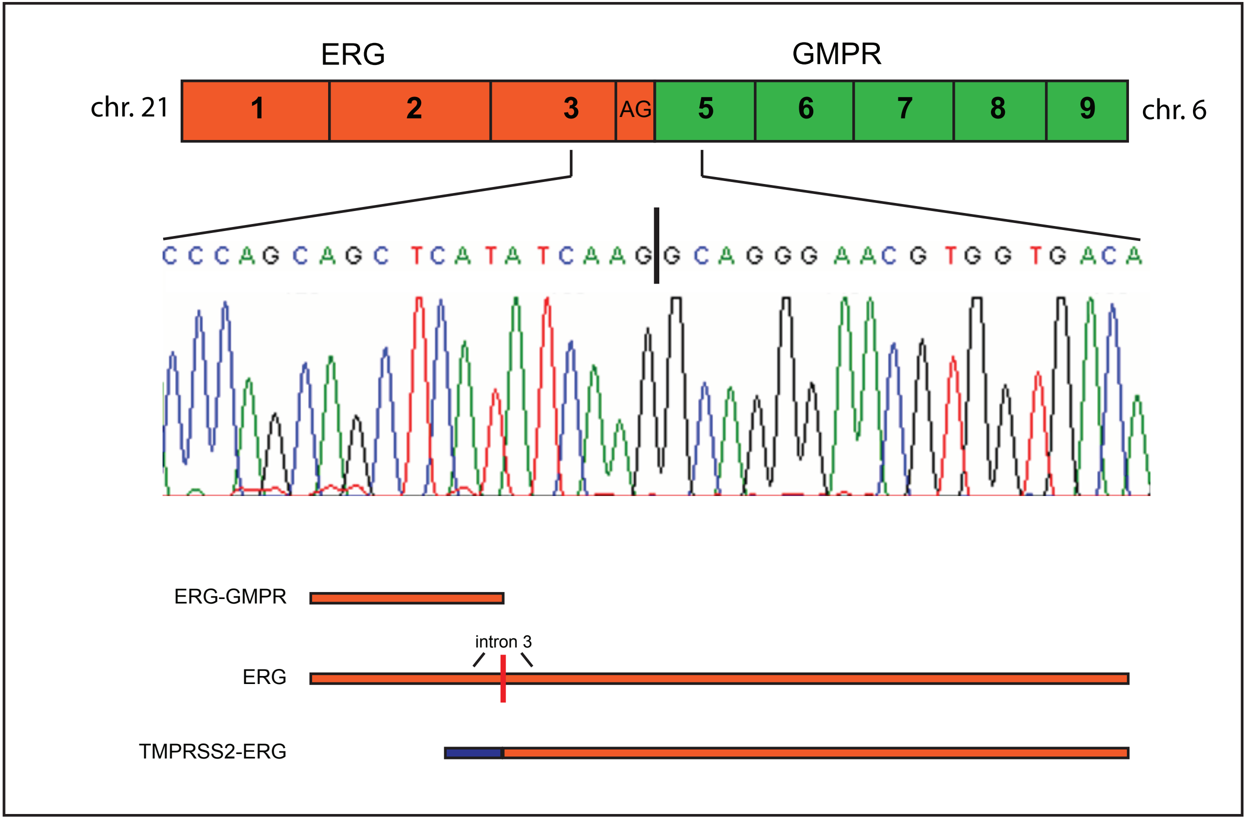

Supplementary Figure 2 Rubin/Gerstein

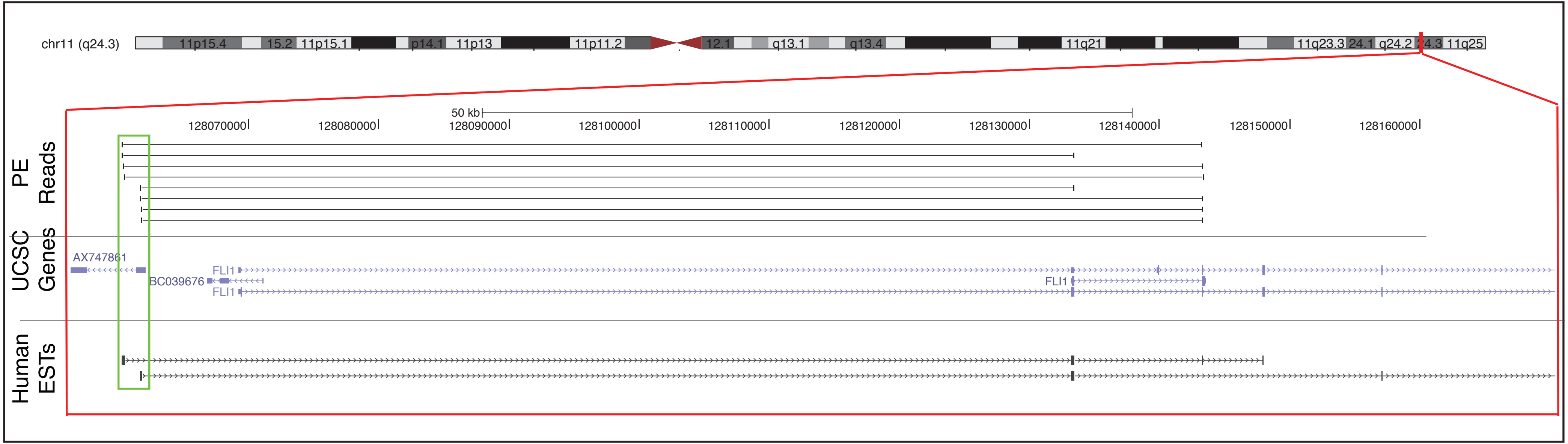

Supplementary Figure 3 Rubin/Gerstein

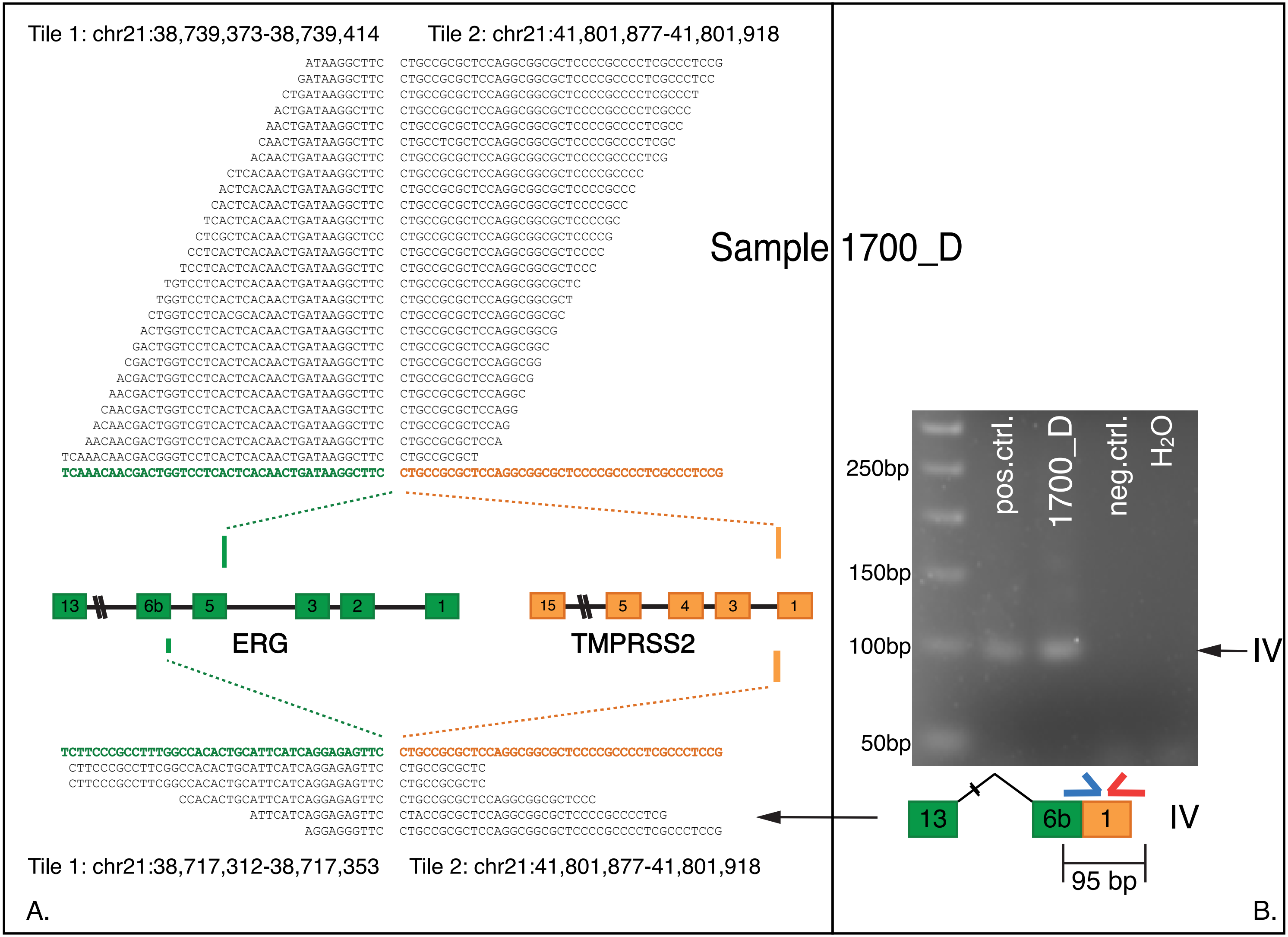

Supplementary Figure 4 Rubin/Gerstein

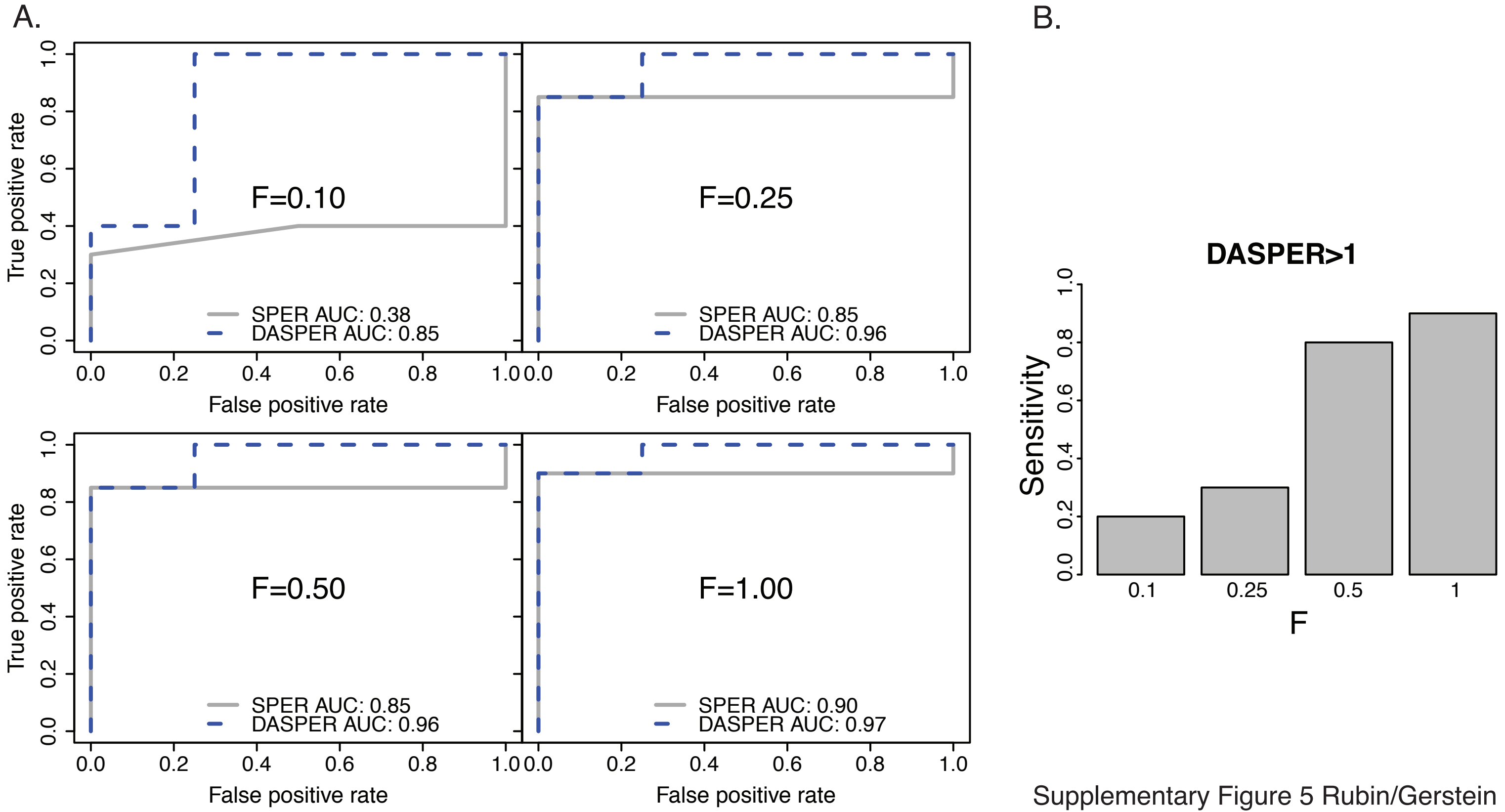

Supplementary Figure 5 Rubin/Gerstein

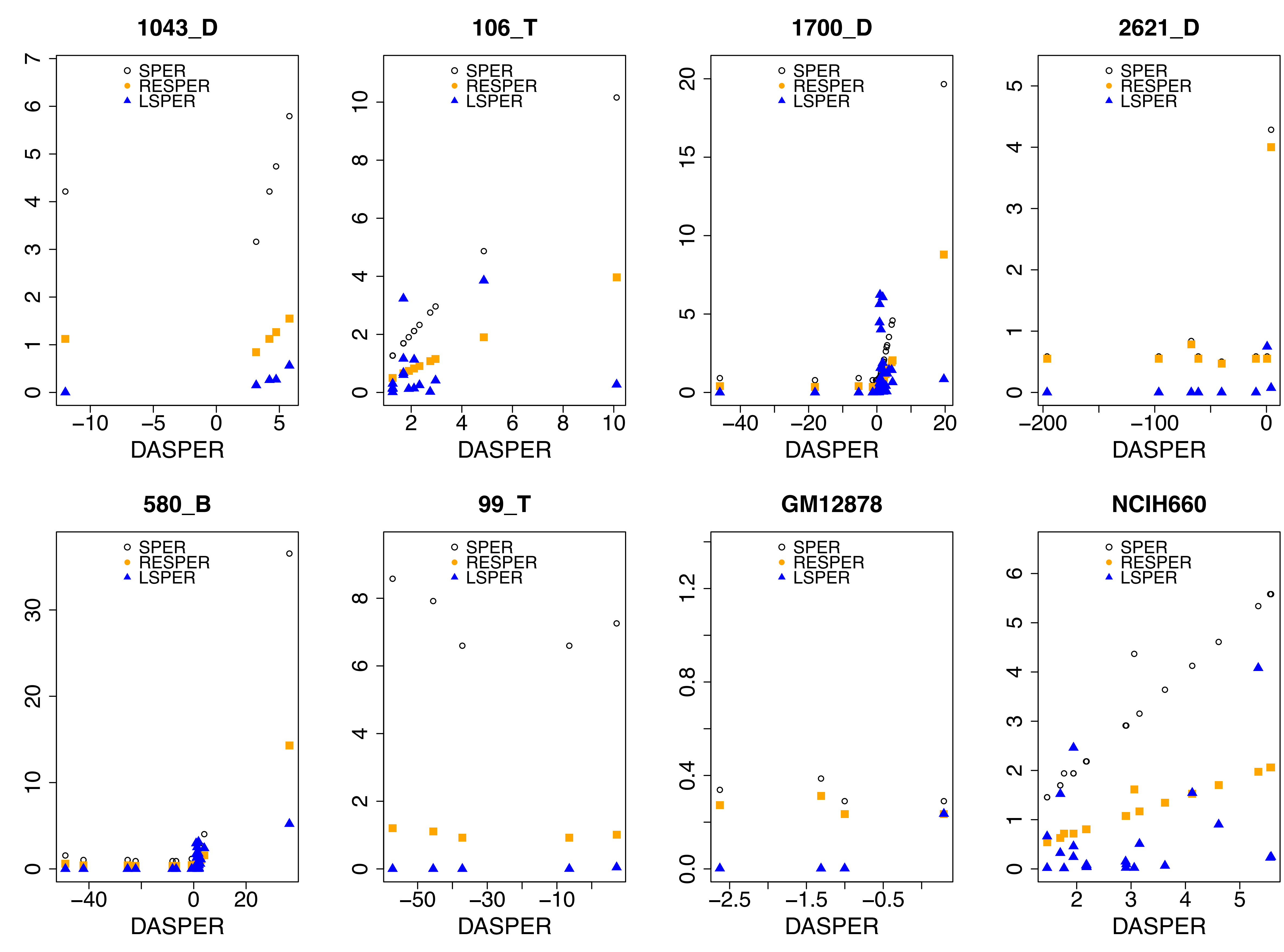

Supplementary Figure 6 Rubin/Gerstein

A.

Identification of potential gene fusions using paired-end reads

Data prefix : [ ]

Minimum number of paired-end reads connecting two genes [5]

Type of gene fusion [All potential gene fusions]

Submit Reset

B.

SPER	DASPER	RESPER	Number of inter paired-end reads	Type	Genomic coordinates	Gene symbol	Description	Genomic coordinates	Gene symbol	Description	
23.558	23.554	36.698	555	intra	chr21:38661052-38955488	ERG	v-ets erythroblastosis virus E26 oncogene like	chr21:41758350-41824913	TMPRSS2	transmembrane protease, serine 2	Details
3.311	3.310	5.158	78	read-through	chr19:57066361-57083016	ZNF577	zinc finger protein 577	chr19:57084299-57100059	ZNF649	zinc finger protein 649	Details
2.419	2.419	3.769	57	read-through	chrY:19493774-19499502	NR_001544	Homo sapiens PRO2834 mRNA, complete cds.	chrY:19553972-19698690	TTY14	Homo sapiens testis transcript Y 14 (TTY14) mRNA, complete cds.	Details

C.

Detailed summary for potential gene fusion candidate

Summary information

Identifier

Number of inter paired-end reads
434

Type : intra

Connected Reads UCSC connectivity graph

Transcript connectivity graph

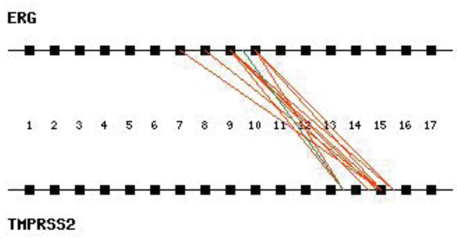

Transcript connectivity table

Pair Type	Entry transcript 1	Entry transcript 2	Counts
exon- exon	7	15	11
exon- exon	8	15	445
exon- exon	9	15	456
exon- exon	10	15	2
exon- intron	9	13	33
exon- intron	9	14	37
exon- intron	9	15	9
exon- intron	10	13	7
exon- intron	10	15	3
exon- boundary	9	left 15	7
intron-intron	9	13	4

Transcript information

Gene symbol(s)	Transcript 1<br>ERG	Transcript 2<br>TMPRSS2
Coordinates	chr21:38661052-38955488	chr21:41758350-41824913
Strand	-	-
Gene description(s)	v-ets erythroblastosis virus E26 oncogene like	transmembrane protease, serine 2
Number of exons	17	17
Number of intra paired-end reads	19429	40218
Links	[UCSC genome browser]<br>[FASTA file]	[UCSC genome browser]<br>[FASTA file]
Expression	[Expression chr21]	[Expression chr21]

Breakpoint analysis

Orientation	Alignments	Breakpoints	
Orientation AB	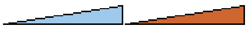 AB	Breakpoints transcript 1<br>UCSC Genome Browser	Breakpoints transcript 2<br>UCSC Genome Browser
Orientation BA	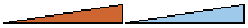 BA	Breakpoints transcript 2<br>UCSC Genome Browser	Breakpoints transcript 1<br>UCSC Genome Browser

Supplementary Figure 7 Rubin/Gerstein

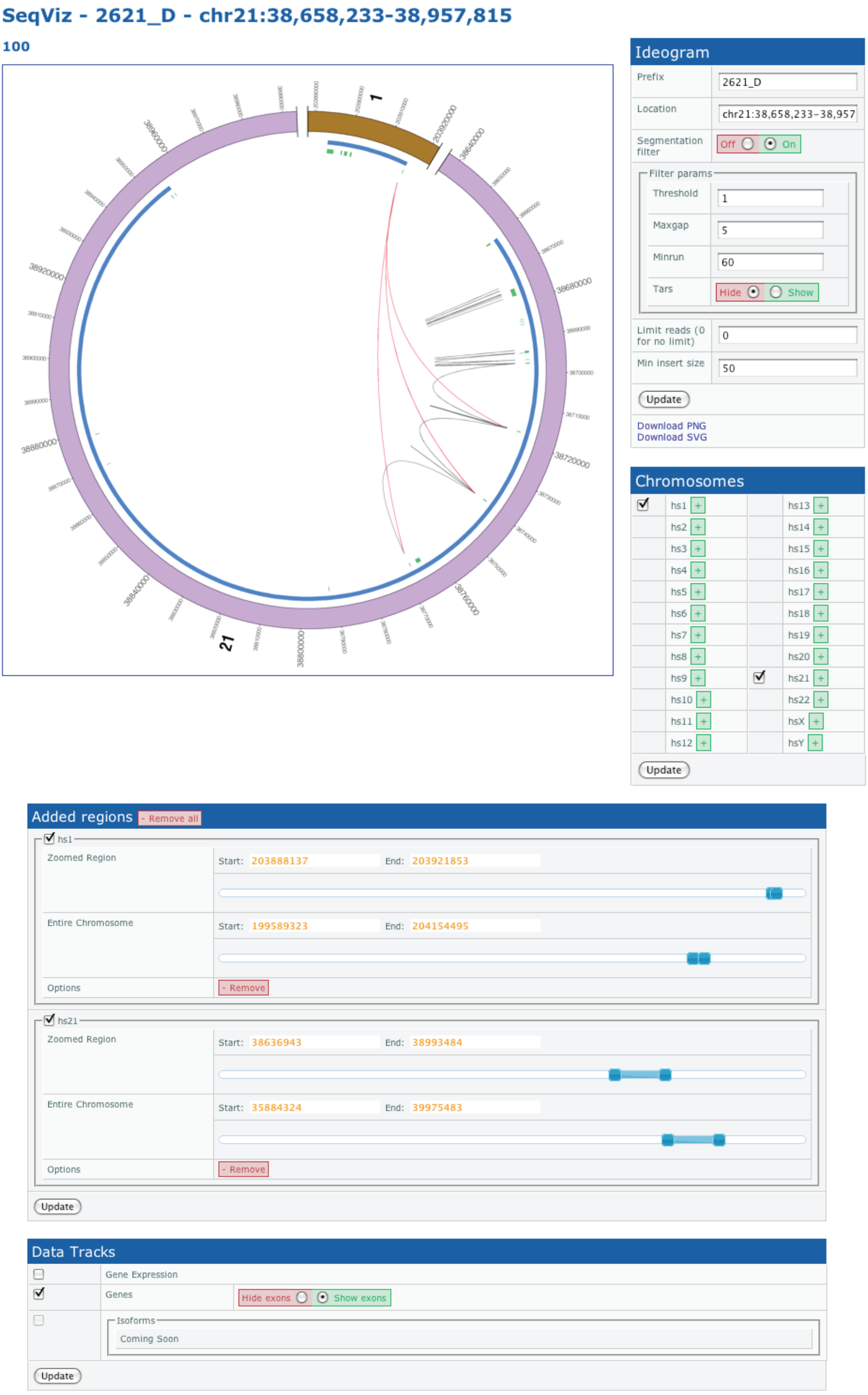

Supplementary Figure 8 Rubin/Gerstein

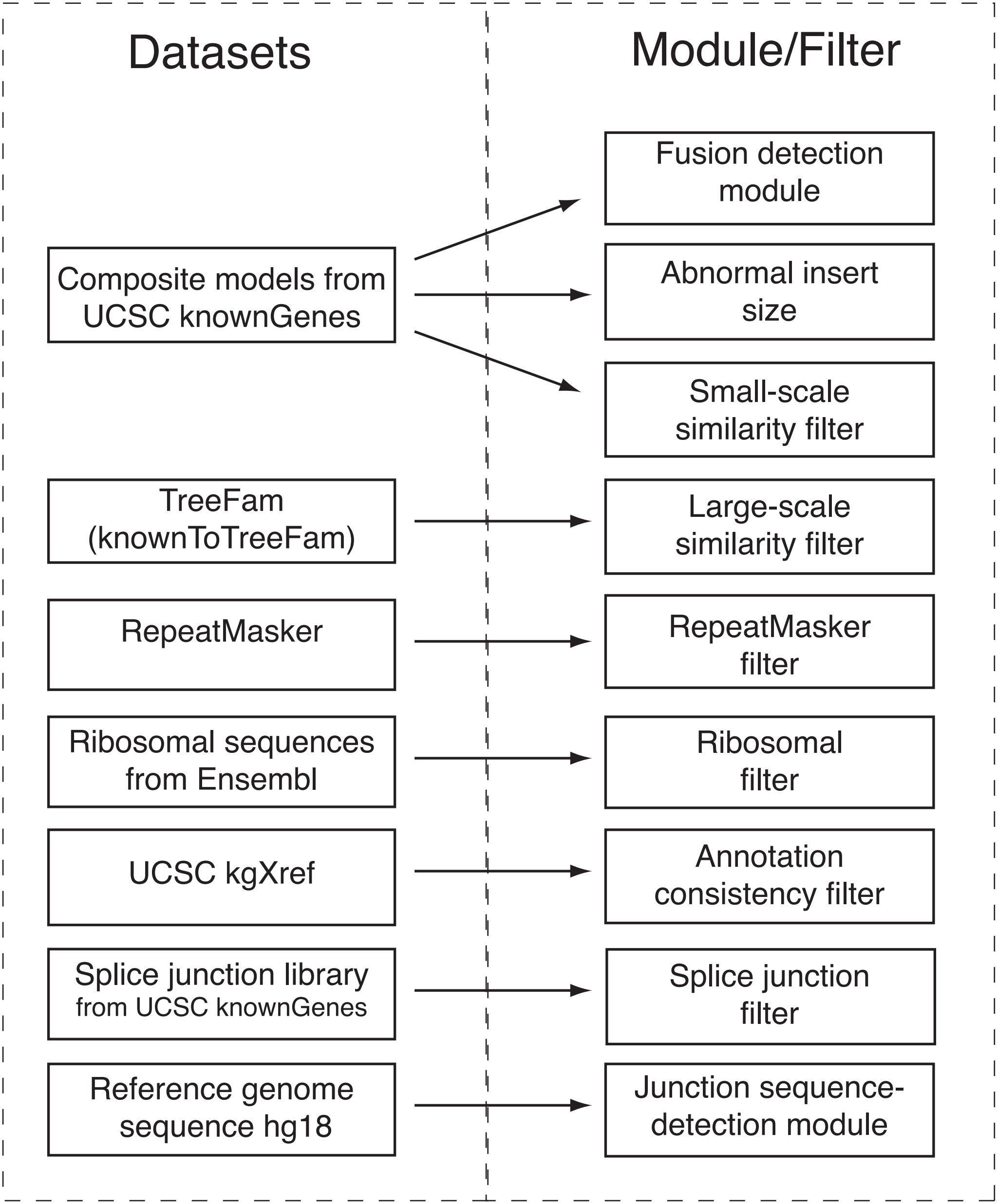

Supplementary figure 9 Rubin/Gerstein
